# Therapeutics Insight with Inclusive Immunopharmacology Explication of Human Rotavirus A for the Treatment of Diarrhea

**DOI:** 10.3389/fphar.2016.00153

**Published:** 2016-06-23

**Authors:** Mohammad Uzzal Hossain, Abu Hashem, Chaman Ara Keya, Md. Salimullah

**Affiliations:** ^1^Department of Biotechnology and Genetic Engineering, Life Science Faculty, Mawlana Bhashani Science and Technology UniversityTangail, Bangladesh; ^2^Microbial Biotechnology Division, National Institute of BiotechnologyDhaka, Bangladesh; ^3^Department of Biology and Chemistry, North South UniversityDhaka, Bangladesh; ^4^Molecular Biotechnology Division, National Institute of BiotechnologyDhaka, Bangladesh

**Keywords:** human rotavirus, diarrhea, pharmacoinformatics, immunoinformatics, vaccine design, drug development

## Abstract

Rotavirus is the most common cause of severe infant and childhood diarrhea worldwide, and the morbidity and mortality rate is going to be outnumbered in developing countries like Bangladesh. To mitigate this substantial burden of disease, new therapeutics such as vaccine and drug are swiftly required against rotavirus. The present therapeutics insight study was performed with comprehensive immunoinformatics and pharmacoinformatics approach. T and B-cell epitopes were assessed in the conserved region of outer capsid protein VP4 among the highly reviewed strains from different countries including Bangladesh. The results suggest that epitope *SU1* (TLKNLNDNY) could be an ideal candidate among the predicted five epitopes for both T and B-cell epitopes for the development of universal vaccine against rotavirus. This research also suggests five novel drug compounds from medicinal plant *Rhizophora mucronata Lamk*. for better therapeutics strategies against rotavirus diarrhea based on 3D structure building, pharmacophore, ADMET, and QSAR properties. The exact mode of action between drug compounds and target protein VP4 were revealed by molecular docking analysis. Drug likeness and oral bioavailability further confirmed the effectiveness of the proposed drugs against rotavirus diarrhea. This study might be implemented for experimental validation to facilitate the novel vaccine and drug design.

## Introduction

Diarrhea caused by rotavirus is accountable for 500,000 deaths with 2.4 million hospitalizations among children under the age of 5 years in every year throughout the world (Parashar et al., 2003, 2006, 2009). It is therefore to be noted that around 85% of them live in developing countries (World Health Organization, 2008). Ruth Bishop identified this virus in 1973 from the small intestine's epithelial cells of children who are affected by diarrhea (Bishop et al., 1974). Humans are mainly infected by species A, B, and C among the available eight species (A, B, C, D, E, F, G, and H) but more than 90% of diarrheal infections occurred by species A. The exact mechanism of rotavirus infection within the public health community is relatively unknown in causing of diarrhea (Rodrigo et al., 2010), particularly in developing countries (Simpson et al., 2007). Rotavirus plays a myriad role in account of 37% of diarrhea related deaths in which 5% occurred < 5 years of age (Tate et al., 2012). It is noticeable that the hospitalization rate of boys is twice than the girls (Ryan et al., 1996; Rheingans et al., 2012). The rotavirus mostly prefer to infect during cool and dry seasons (Levy et al., 2009; Atchison et al., 2010). Rotavirus infection shows the climate variability that peaks in winter to cause diarrhea. Comparatively, less infection rate was recognized in temperate regions like United States (Glass et al., 1996), Japan (Konno et al., 1983; Nakagomi et al., 2005), northern Asian regions (Bresee et al., 2004), temperate regions in Australia (Bishop et al., 2001), and Europe (Cook et al., 1990). But, the southern Asian regions like Bangladesh is recognized as fertile area to infection of rotavirus as it has the tendency to occur all year round (Stoll et al., 1982). In 1981, Colorado was badly affected by an outbreak of rotavirus A through contaminated municipal water (Hopkins et al., 1984). In 2005, Rotavirus was responsible for the largest epidemic of diarrhea in Nicaragua, associated with mutations in the rotavirus A genome, thereby escaping the virus release to the prevalent immunity in the population (Bucardo et al., 2007). In 1977, a similar large outbreak was also appeared in Brazil (Linhares et al., 1981).

Rotaviral genome, shelled with three protein (a core, inner membrane, and outer capsid), contains 11 segmented gene (double stranded; Gentsch et al., 1996). The both structural protein (VP1–VP4, VP6, VP7) and non-structural protein (NSP1–NSP4) are encoded by rotaviral each RNA gene segment, but only gene segment 11 code for NSP5 and NSP6. The outer shell that is used to characterize the rotavirus strain, formed by VP7 (a glycoprotein-G protein) and VP4 (a protease-cleaved protein—P protein). The combination of G and P proteins is mediated by VP4 and VP7 structural protein due to its independent nature of segregation both *in vivo* and *in vitro*. And more than 80% of severe rotavirus disease is caused by the mostly common four combinations (P[8]G1, P[4]G2, P[8]G3, P[8]G4) of G and P proteins (Matthijnssens et al., 2009). It is suspected that clinical protection probably involves mucosal (intestinal), systemic antibody response, and cell-mediated immunity however it is difficult to realize the mechanisms, protection as well as duration of protection against rotavirus infection (Santos and Hoshino, 2005; Kang et al., 2009).

Vaccinomics is the integration of immunogenetics and immunogenomics with bioinformatics for the development of vaccines (Poland et al., 2009). This method has been exclusively used for the development of new vaccines based on the bioinformatics. With the current advancement in the genome sequencing and protein sequence databases, the rapid *in silico* informatics-based approach have obtained huge acceptance over the traditional time consuming process (Flower, 2008). For combating a number of life-threating diseases like multiple sclerosis (Bourdette et al., 2005), malaria (López et al., 2001), and tumors (Knutson et al., 2001) vaccinomics already demonstrated its potentials.

To mitigate the threat of re-infection of diseases both humoral and cell mediated immune response can play crucial role in this perspectives. It is therefore necessary for an ideal vaccine to stimulate a specific immunological response (Atanas and Irini, 2013). In this case, the identification of B-cell and T-cell epitope can be effective to elicit immune response which can further reinforce the possibility of novel vaccine design.

Computational tools and software, and drug–protein simulation analysis have become very dependable and powerful methods in the drug designing studies. These analysis can significantly contribute to find out exclusive binding sites as well as to determine suitable inhibitors to the target proteins, respectively. Inhibitor molecules of envelope protein in dengue virus were successfully identified by utilizing such types of methodology (Yennamalli et al., 2009).

In our study, for the prediction of an ideal candidate for vaccine development we have identified the conserved region of the VP4 protein of Human rotavirus A and suggested a potential T-cell and B-cell epitope by immunoinformatics approach. Furthermore, drug discovery approach has also been conducted to identify the most suitable drug compounds and to stimulate the inhibition of the target site by natural medicinal plant compounds using computational methods. This study has been conducted with an aim to facilitate further laboratory research for the complete treatment and prevention of diarrhea caused by Human rotavirus A.

## Materials and methods

**Figure 1:**
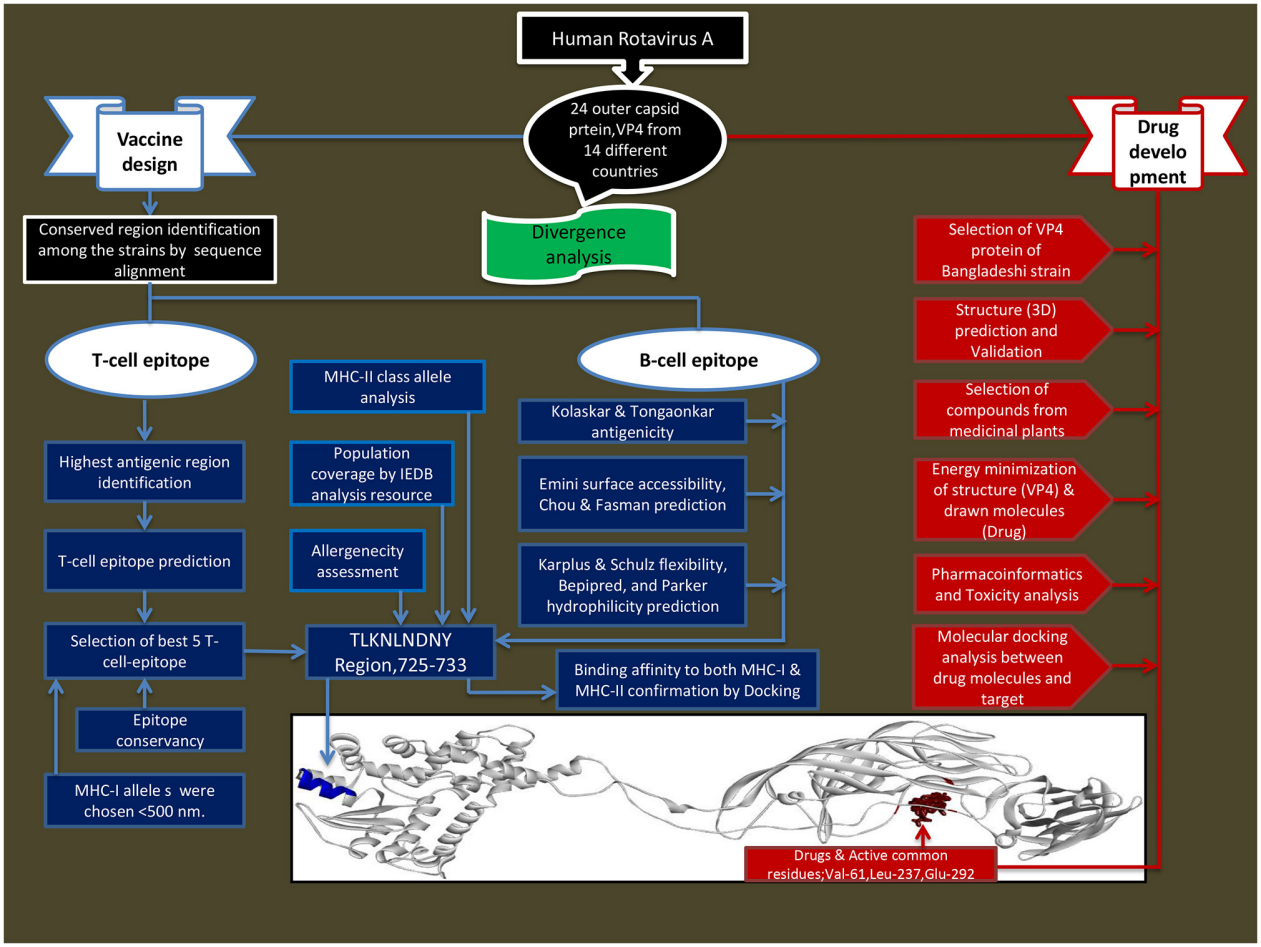
**Schematic presentation of vaccine and drug designed against human rotavirus**.

The outline of this study was shown in Figure 1.

### Retrieving VP4 protein sequences of human rotavirus A

Twenty-four outer capsid VP4 protein of human rotavirus A of different strains from 14 different countries specifically Bangladesh, United States, United Kingdom, Australia, Italy, Belgium, Venezuela, Israel, Philippines, Thailand, Sweden, Japan, Indonesia, and India were retrieved from UniProt Knowledge Base (UniProtKB) database (http://www.uniprot.org; Apweiler et al., 2004; The UniProt Consortium, 2014) and NCBI (http://www.ncbi.nlm.nih.gov/). The retrieved sequences, which are highly reviewed and manually annotated, were then stored in FASTA format for the immunoinformatics and pharmacoinformatics elucidation.

### Divergence analysis and conserved region identification

CLC Sequence Viewer v7.0.2 (http://www.clcbio.com) from CLC drug discovery workbench was used for the analysis of the divergence among the different strains of the outer capsid protein VP4.BioEdit v7.2.3 sequence alignment editor (Hall, 1999) was used through multiple-sequence alignment (MSA) with ClustalW (Thompson et al., 1994) to identify conserved sequences of different strains.

### Identification of protein disorder region

Globplot 2.3 (http://globplot.embl.de/) was used to identify the protein disorder region of the VP4 protein of human rotavirus A.

### Structure analysis of VP4 protein

The automated protein modeling program MODELLER 9v11 (Šali et al., 1995) through HHpred (Söding, 2005; Söding et al., 2005) was used to predict the 3D structure of VP4 protein by satisfying spatial restraints. The evaluation tools ProCheck (Laskowski et al., 1993) and Verify3D (Eisenberg et al., 1997) were applied to assess the predicted three-dimensional model of VP4 protein of Human Rotavirus A.

### Vaccine design

#### Determination of highest antigenic conserved region

The antigenicity of the conserved region was determined by the VaxiJen v2. 0 (Doytchinova and Flower, 2007) server in which the default parameters was used for the identification.

#### Prediction of T cell epitope

To identify cytotoxic T lymphocyte (CTL) epitopes from the most antigenic conserved region a server entitled NetCTL 1.2 (http://www.cbs.dtu.dk/services/NetCTL/), was used which is based on neural network architecture.

The possible epitopes were predicted by the *in vivo* peptides processing which utilize 12 HLA-I super types (A1, A2, A3, A24, A26, B7, B8, B27, B39, B44, B58, and B62; Larsen et al., 2005). To generate more epitopes from the conserved region the threshold value 0.5 was used to maintain specificity as well as sensitivity. The epitopes were considered for the vaccine development based on total high score combining from MHC class I binding, transporter of antigenic peptides (TAP) transport efficiency, and proteosomal cleavage. Stabilized Matrix Method (SMM; Peters and Sette, 2005) was employed to determine the half-maximal inhibitory concentration (IC50) values of peptides to the binding on MHC-I molecules. A web-based tool was used to predict proteasomal cleavage, TAP transport, and MHC-I for the selected epitopes (Tenzer et al., 2005). The TAP transport, MHC-I binding and proteasomal processing produce an overall score for each individual peptidases a T cell epitope. SMM was also implemented for the prediction of MHC-II molecules that cover the best candidates epitope from the conserved region.

#### Analyzing epitope conservancy

IEDB analysis resource (Bui et al., 2007) was used to analyze the epitope conservancy for each individual predicted epitopes. The given protein sequences were searched in this web based tool for identities to calculate the conservancy level of all predicted epitopes.

#### Population coverage prediction

IEDB population coverage calculation tool (Bui et al., 2006) was used for the selection of population coverage of each individual epitope. To predict the population coverage for the corresponding epitope the allelic frequency of the interacting HLA alleles were used.

#### Design of epitope structure

PEP-FOLD (Thevenet et al., 2012) server, which is a *de novo* approach designed to predict peptide structures from amino acid sequences was employed to build the three dimensional (3D) structure of the highest conserved T-cell epitope *SU1*. Consequently, the best model was selected from the proposed five model to analyze the interactions with HLAs (HLA-I and HLA-II).

#### Docking simulation study

AutodockVina (Trott and Olson, 2010) was employed to observe the binding affinity between proposed interacting HLA molecules and *SU1* epitope. HLA-B^*^15:01 from HLA-I and HLA-DR from HLA-II were selected for the binding analysis. The 3D crystal structure of HLA-B^*^15:01 named 1XR8 and HLA-DR named 1D5M were retrieved from RCSB (Research Collaboratory for Structural Bioinformatics) database (Berman et al., 2005). Before performing the docking study, 1XR8 and 1D5M were subjected to Discovery studio (Van Joolingen et al., 2005) to remove the ligand which made complex with HLA molecules.

#### Identification of the B cell epitope

Identification of the probable immunogenic epitopes in a antigenic protein sequence could offer the reduction of wet lab analysis for vaccine study. To initiate an immune response B-cell epitope plays a crucial role by interacting with the B lymphocytes (Nair et al., 2002). IEDB tools were utilized to identify essential properties of the B cell antigenicity, including the Kolaskar and Tongaonkar antigenicity scale (Kolaskar and Tongaonkar, 1990), Emini surface accessibility prediction (Emini et al., 1995), Karplus and Schulz flexibility prediction (Karplus and Schulz, 1985), Bepipred linear epitope prediction analysis (Larsen et al., 2006), and Chou and Fasman beta turn prediction analysis (Chou and Fasman, 1978; Rini et al., 1992; Yamin et al., 2002). The hydrophilicity of the predicted epitopes was also analyzed by utilizing the Parker hydrophilicity prediction tool (Parker et al., 1986) of Immune Epitope Database (IEDB) in which default threshold was used.

### Drug development

#### Selection of medicinal compounds

Primarily, literatures were searched in NCBI (http://www.ncbi.nlm.nih.gov), Pubmed (www.ncbi.nlm.nih.gov/pubmed), and Google Scholar (http://scholar.google.com/) for the identification of effective medicinal plant compounds that had been comprehensively validated with lab experiment.

#### Preparation of drug molecules

The PDB file of Caffeic acid, Stigmast-7-en-3β-ol, and Quercetin were retrieved from Pubchem (http://www.ncbi.nlm.nih.gov/pccompound/) and Zinc database (http://zinc.docking.org/). But later two drug compounds were not available in those databases. Therefore, they were designed by using ACD/Chemsketch (Yamin et al., 2002) according to their structure and then generated their structural data file (SDF) and Mol file. The Molfile of Rhizophorine, 1-Hydroxy-5-oxobicyclo [6.4.0] dodecane were converted to PDB file by using Open Babel (O'Boyle et al., 2011). Afterwards, the 3D structure optimization of these compounds was done by the ACD/Chemsketch (Yamin et al., 2002).

#### Energy minimization of projected model and drawn molecules

Energy minimization of predicted 3D model and drawn structure of medicinal drug compounds were calculated for the accurate alignment which is a key requirement to link up the structural gap between template and target. Encompass loop rearrangements, secondary structure elements, and repacking of core residues with YASARA (Yet Another Scientific Artificial Reality Application) force field was performed for calculating the relative binding free energies of homology model and drawn structures (Krieger et al., 2009). The parameters of YASARA are employed for the minimization of damage occurred in predicting homology model and drawn structures with the full atomic description. The YASARA energy functions are known to be essential for protein stability (Sippl, 1993; Vriend and Sander, 1993; Kuszewski et al., 1997).

#### QSAR (quantitative structure activity relationship) studies

Quantitative structure activity relationship (QSAR) was calculated for the effective drug nature properties. The structural properties from QSAR were retrieved from Click2drug (http://www.click2drug.org/), Zinc database (http://zinc.docking.org/), Osiris property explorer (Sander, 2001), Molinspiration (Mishra and Raghava, 2011), and AcTor (Judson et al., 2008).

#### Docking analysis

The energy minimized homology model and the medicinal compounds were then performed in Autodock 4.2 (Morris et al., 2009) and AutodockVina (Trott and Olson, 2010) for confirming the better docking analysis into the binding site. Before initiating the docking study, all water molecules were removed and polar hydrogen were added into minimized model structure. AutoDock tools 1.5.6 was used to prepare the input pdbqt file for energy minimized envelope glycoprotein. A grid box parameter were set in size with 40 × 40 × 40 points and center with 168.948 × 349.143 × 240.353, and 0.5°A for grid spaces well. Then AutodockVina was used for all docking runs. The same parameters for identifying the crucial amino acid interactions with drug compounds were used for docking runs in Autodock 4.2. In Autodock 4.2 contrast to AutodockVina, Kollman united atom charges (Duan et al., 2003) were added to the model structure. Autogrid was used to generate the ligand map files for search space to comprise the ligand binding site. To generate the set of possible conformation Lamarckian Genetic Algorithm (LGA), which is well thought-out as one of the best for small molecule docking studies, was used in Autodock 4.2. The initial population size 150 was performed for the every docking runs. The conformation was preferred with the lowest docked energy after the docking interaction between target built model and inhibitors. The three binding energy terms: intermolecular energy, internal energy of ligand, and torsional free energy were generated by Autodock 4.2. Afterwards, the final docked energy or binding affinity was calculated from the sum of intermolecular energy and internal energy of ligand. The molecular visualization of protein-ligands were analyzed by PyMol (Seeliger and de. Groot, 2010), Yasara (Krieger et al., 2009), RasMol (Roger and James, 1995), UCSF Chimera (Pettersen et al., 2004), and Discovery studio (Van Joolingen et al., 2005).

#### Analyzing active site

Computed Atlas of Surface Topography of proteins (CASTp; Dundas et al., 2006) was used to identify the active regions which further allowed to recognize crucial amino acids from drug-protein interactions. This method allows to determine the active residues, active sites, binding sites, and internal cavities of protein.

#### Pharmacoinformatics studies

The pharmacophore properties of our selected plant medicinal drug molecules were carried out by the online-based and license-agreed software. Absorption, distribution, metabolism, excretion, and toxicity (ADME/Tox) confer the kinetics of drug exposure and nature to the tissues and pharmacological activity of the compounds. The Osiris property explorer (Sander, 2001), Molinspiration (Mishra and Raghava, 2011), ACToR (Aggregated Computational Toxicology Resource; Judson et al., 2008), admetSAR (absorption, distribution, metabolism, excretion, and toxicity Structure-Activity Relationship database; Cheng et al., 2012), and ACD/I-lab (Masunov, 2001) were utilized for the calculation of ADME properties and toxic profile. The quantitative drug likeness were calculated by the DruLiTo software tools (Bickerton et al., 2012).

## Results

### Analysis of the retrieved sequences (divergence and conserved region)

**Figure 2:**
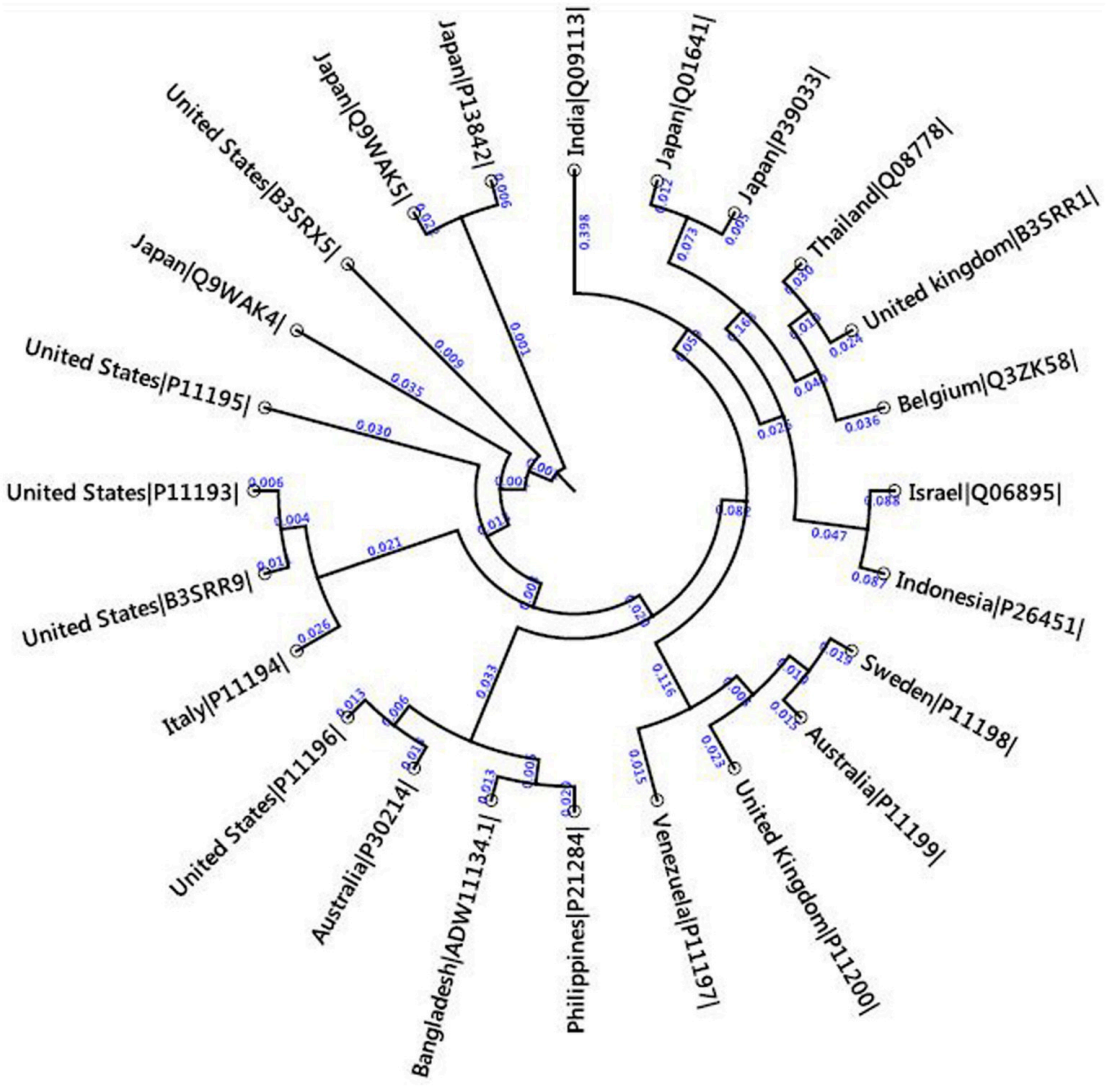
**Divergence analysis of 24 VP4 proteins from 14 different countries**. The neighbor joining method was used for phylogenetic tree construction for iteratively joining clusters which are close to each other but at the same time far from all other clusters. In this method, “Kimura” protein distance measurement was selected for the equal amino acid frequency and equal substitution rates. Bootstrap value was also shown by producing 1000 replicates for measuring the accurate distance among the 24 strains.

**Figure 3:**
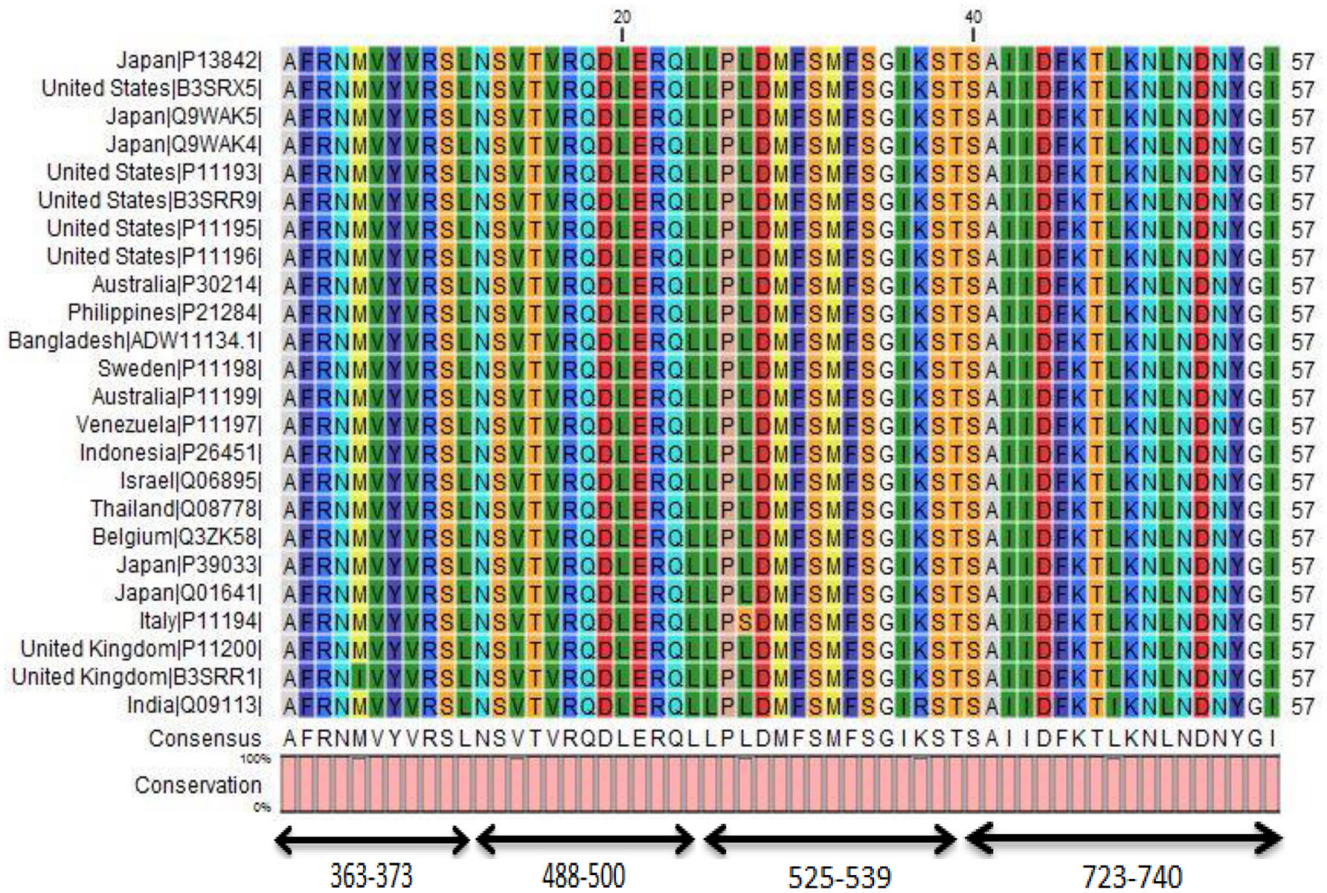
**Conserved region in 24 different strains of Human Rotavirus A**. Four conserved regions of more than 10 amino acids were found on multiple sequence alignment. The conserved region “SAIIDFKTLKNLNDNYGI” showed higher antigenic score which allowed to predict some probable candidates for peptide based vaccine design.

**Figure 4:**
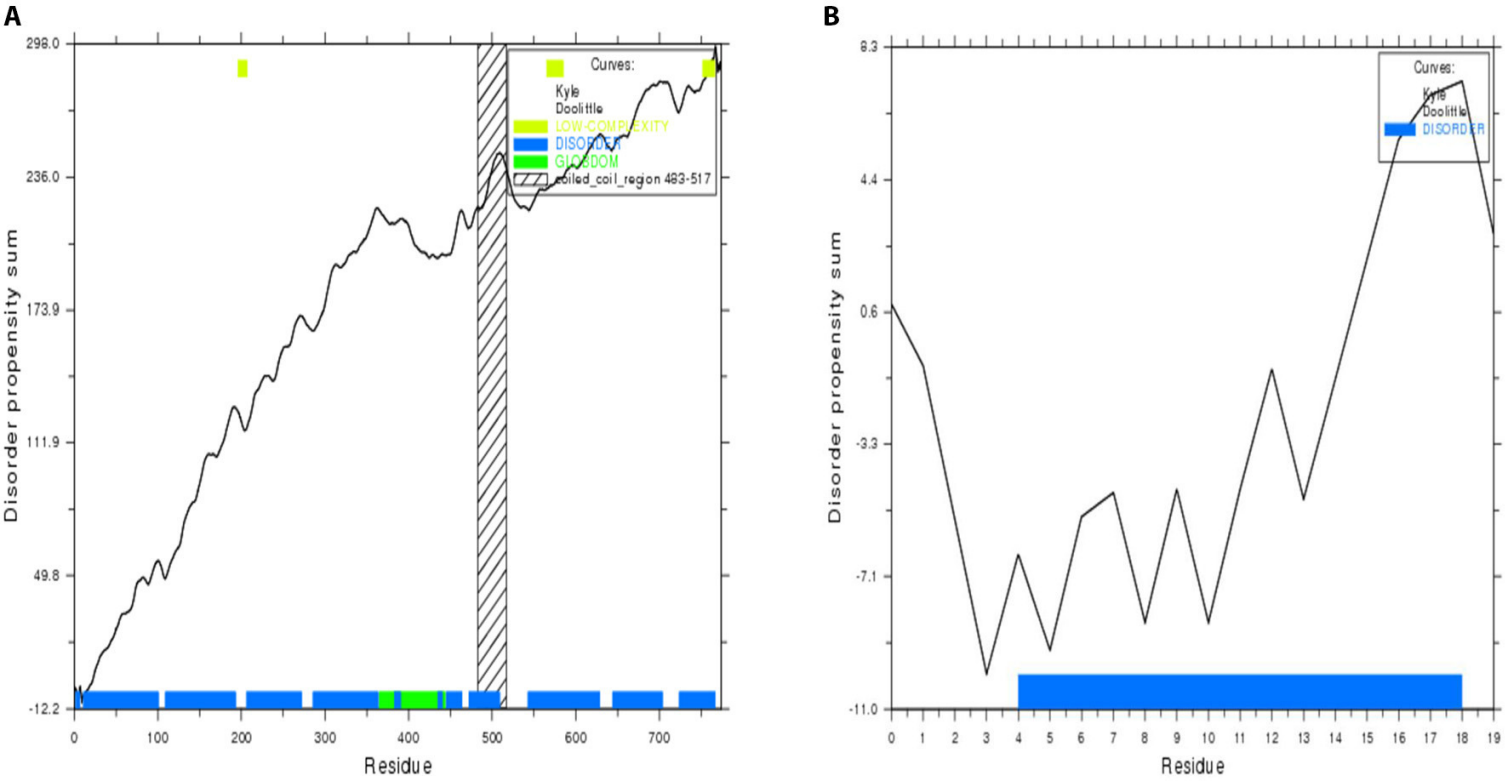
**Protein disorder regions of VP4 protein including the most antigenic conserved region. (A)** The disorder regions of the whole protein sequence. **(B)** The disorder region in the conserved region “SAIIDFKTLKNLNDNYGI.” Here, blue color indicates the disorder region while green color indicates the globular domain.

The 24 outer capsid VP4 protein of Human rotavirus A from 14 countries were retrieved and analyzed for their evolutionary relationship. The results suggest us that they are very similar in distance (Figure 2) analyzed by Node type (Root, Internal node, and Leaf), Branch length, and Bootstrap values (Internal Node) of 24 strains of rotavirus (Table S1). Four conserved regions of more than 10 amino acids were identified from the multiple sequence alignment of 24 retrieved VP4 proteins (Figure 3, Table 1, and Figure S1). Among the conserved regions, no. 2 and 4 included in all the 24 strains tested, while region 1 and 3 included 23 except UK and Italy, respectively. Thereafter, the result from globplot 2.3 identified the several disease causing region whereas conserved region SAIIDFKTLKNLNDNYGI was also one of them (Figure 4).

**Table 1:** **Conserved region of VP4 from MSA and their antigenic score**.

**Conserved region no**.	**Amino acid sequence**	**Position of conserved regions amino acids**	**Vaxijen score (threshold 0.4)**
1	AFRNMVYVRSL	363–373	0.2661
2	NSVTVRQDLERQL	488–500	−0.2378
3	LPLDMFSMFSGIKST	525–539	0.3204
4	SAIIDFKTLKNLNDNYGI	723–740	0.9606

### Homology modeling and validation

The 3D structure of VP4 protein (in this case Bangladeshi strain, as all sequences are mostly similar) was predicted by using the Modeller 9v11 displayed in Figure S2. Different validation tools were employed for the structural quality assessment. The Ramachandran plot from Procheck identified 88.6% residues in most favored regions (Figure S3). VERIFY3D also ensure a good 3D structure of VP4 protein of rotavirus shown in Figure S4.

### Antigenicity determination of conserved region

The conserved region having amino acid composition “SAIIDFKTLKNLNDNYGI” was identified as the highest antigenicity among the entire conserved region by multiple sequence alignment (Table 1). This region attainted the highest predicted antigenic score accounts to 0.9606.

### T cell epitope identification

NetCTL server was implemented to find T-Cell epitopes on the conserved region of VP4 protein. The server recognizes probable overlapping epitopes on the selected protein sequence by utilizing neural network architecture and gives a combinatorial score by predicting peptide MHC class-I binding based on all MHC class-II super types, proteasomal C terminal cleavage and TAP transport efficiency collectively. It could allow to identify five epitopes TLKNLNDNY, AIIDFKTLK, SAIIDFKTL, IDFKTLKNL, and KNLNDNYGI based on the highest combinatorial score (Table 2). The set of enzymes consisting proteasomal cleavage have the capability to cleave the proteins to form smaller peptides. These digested peptides are identified by MHC class 1 molecules and then MHC class 1 forms a complex with these digested peptides and transported to the endoplasmic reticulum, a process further assisted by transport associated proteins (TAP) before being presented to the T-cells on the plasma membrane of the cell. MHC class 1 binding prediction tool from IEDB identified a number of MHC class 1 molecule where selected five epitopes were employed. The principle of this method is based on stabilized matrix method (SMM) that allow to select HLA molecules to predicted epitopes utilizingIC50 nM unit. In our study, IC50-value less than 500 nm (IC50 < 500) was considered for the selection of the MHC-I molecules which ascertained higher affinity to the epitopes (Table 3). Every single epitope has individual overall score generated from MHC-I processing efficiency tool accordingly to the proteasomal cleavage efficiency, TAP transport efficiency, and MHC-I binding efficiency. A higher combined score of the peptide tells us that they are well-processed for presentation which ensures to induce an effective immune response (Table 3). An effective immune response is determined by the successful recognition of epitopes by HLA molecules with strong affinity. So, a peptide can prompt better immune response if it is interacted with more HLA alleles. The 9-mer epitope TLKNLNDNY showed the highest affinity for 11 MHC-1 molecules including HLA-C^*^03:03(−0.14), HLA-B^*^15:01(−0.13), HLA-A^*^30:02(−0.08), HLA-B^*^40:13(0.17), HLA-C^*^14:02(0.31), HLA-B^*^15:02(0.43), HLA, A^*^32:15(0.90), HLA-A^*^68:23(1.08), HLA-B^*^27:20(1.10), HLA-A^*^32:07(1.28), HLA-C^*^12:03(1.61) among the five selected epitopes. As the conserved epitopes have the greater ability to induce strong immunization, epitope conservancy was analyzed to the possible all VP4 proteins available in databases (both reviewed and unreviewed VP4 proteins; Table 3). The epitope TLKNLNDNY showed the highest 65.66% conserveness whereas the rest of four epitopes AIIDFKTLK, SAIIDFKTL, IDFKTLKNL, and KNLNDNYGI showed 65.36, 65.36, 65.36, and 63.65% conservancy, respectively. On the basis of number of MHC-1 molecules and epitope conservancy TLKNLNDNY was selected to identify the MHC-II molecules (Table 4).

**Table 2:** **The selected epitopes, on the basis of their overall score predicted by the NetCTL server**.

**Number**	**Epitopes**	**Overall score (nm)**
*SU1*	TLKNLNDNY	1.0488
*SU2*	AIIDFKTLK	1.0269
*SU3*	SAIIDFKTL	0.5535
*SU4*	IDFKTLKNL	0.4882
*SU5*	KNLNDNYGI	0.3519

**Table 3:** **The five potential T cell epitopes, along with their interacting MHC-I alleles and total processing score, and epitope conservancy result from the highest antigenic conserved peptide**.

**Number**	**Epitopes**	**Interacting MHC-I allele with an affinity of, < 500 nM and the score of percentile rank**	**Epitope conservancy rate (%)**
*SU1*	TLKNLNDNY	HLA-C*12:03(1.61)	65.66
		HLA-A*32:07(1.28)	
		HLA-B*27:20(1.10)	
		HLA-A*68:23(1.08)	
		HLA-A*32:15(0.90)	
		HLA-B*15:02(0.43)	
		HLA-C*14:02(0.31)	
		HLA-B*40:13(0.17)	
		HLA-A*30:02(−0.08)	
		HLA-B*15:01(−0.13)	
		HLA-C*03:03(−0.14)	
*SU2*	AIIDFKTLK	HLA-A*11:01(0.14),	65.36
		HLA-C*12:03(0.01),	
		HLA-A*32:07(−0.02),	
		HLA-C*03:03(−0.17),	
		HLA-A*68:23(−0.26),	
		HLA-B*27:20(−0.68),	
		HLA-A*03:01(−0.78),	
		HLA-A*68:01(−0.85)	
*SU3*	SAIIDFKTL	HLA-C*03:03(1.63),	65.36
		HLA-A*68:23(1.09),	
		HLA-A*32:07(0.97),	
		HLA-C*12:03(0.87),	
		HLA-B*27:20(0.54),	
		HLA-B*40:13(0.52),	
		HLA-A*02:50(0.52),	
		HLA-C*15:02(0.49),	
		HLA-A*32:15(0.44),	
		HLA-B*15:02(0.08),	
		HLA-A*02:17(−0.01)	
*SU4*	IDFKTLKNL	HLA-A*02:50(1.14),	65.36
		HLA-B*27:20(1.05),	
		HLA-B*40:13(1.01),	
		HLA-C*12:03(0.71),	
		HLA-A*68:23(0.69),	
		HLA-A*32:07(0.61),	
		HLA-A*02:17(0.31),	
		HLA-C*14:02(0.28),	
		HLA-B*15:02(0.17),	
		HLA-C*03:03(−0.05),	
		HLA-B*40:02(−0.41)	
*SU5*	KNLNDNYGI	HLA-A*32:07(0.42),	63.65
		HLA-A*02:50(0.36),	
		HLA-B*40:13(0.30),	
		HLA-B*27:20(−0.02),	
		HLA-A*68:23(−0.37),	
		HLA-A*32:01(−0.42),	
		HLA-C*12:03(−0.44),	
		HLA-A*32:15(−0.68),	
		HLA-C*15:02(−0.95),	
		HLA-C*03:03(−1.00),	
		HLA-A*02:17(−1.09)	

**Table 4:** **MHC–II molecules from the desired peptide epitope**.

**Epitope peptide**	**MHC-II molecules**
*SU1*	HLA-DRB1*13:02
	HLA-DRB1*12:01
	HLA-DRB1*03:01
	HLA-DRB3*01:01
	HLA-DQA1*01:01/DQB1*05:01
	HLA-DRB4*01:01
	HLA-DQA1*05:01/DQB1*02:01
	HLA-DQA1*03:01/DQB1*03:02
	

**Figure 5:**
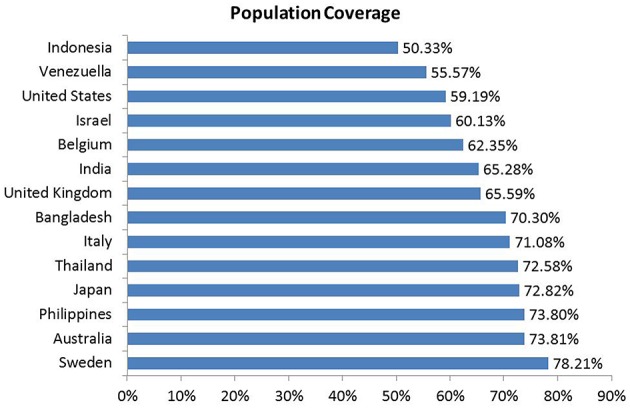
**Population coverage of different strains of VP4 protein**. The peptides with different HLA binding specificities will increase the population coverage as the various HLA types are expressed at radically different frequencies in various ethnicities. The populations were grouped upon 14 countries for our study.

Population coverage is the measurement between people in a given area and both (MHC-I and MHC-II) alleles of the query epitopes. The 14 different countries were considered for the analysis of population coverage by the IEDB's population coverage tool that generated 70.30% coverage to the Bangladeshi people whereas highest 78.21% population coverage was found in the Sweden. The epitopes indicated that 65.59–78.21% coverage in Europe whereas in American region it was found 55.57–59.19% population coverage. Moreover, Asian region showed 50.33–73.80% cumulative population coverage. The results are shown in Figure 5.

**Figure 6:**
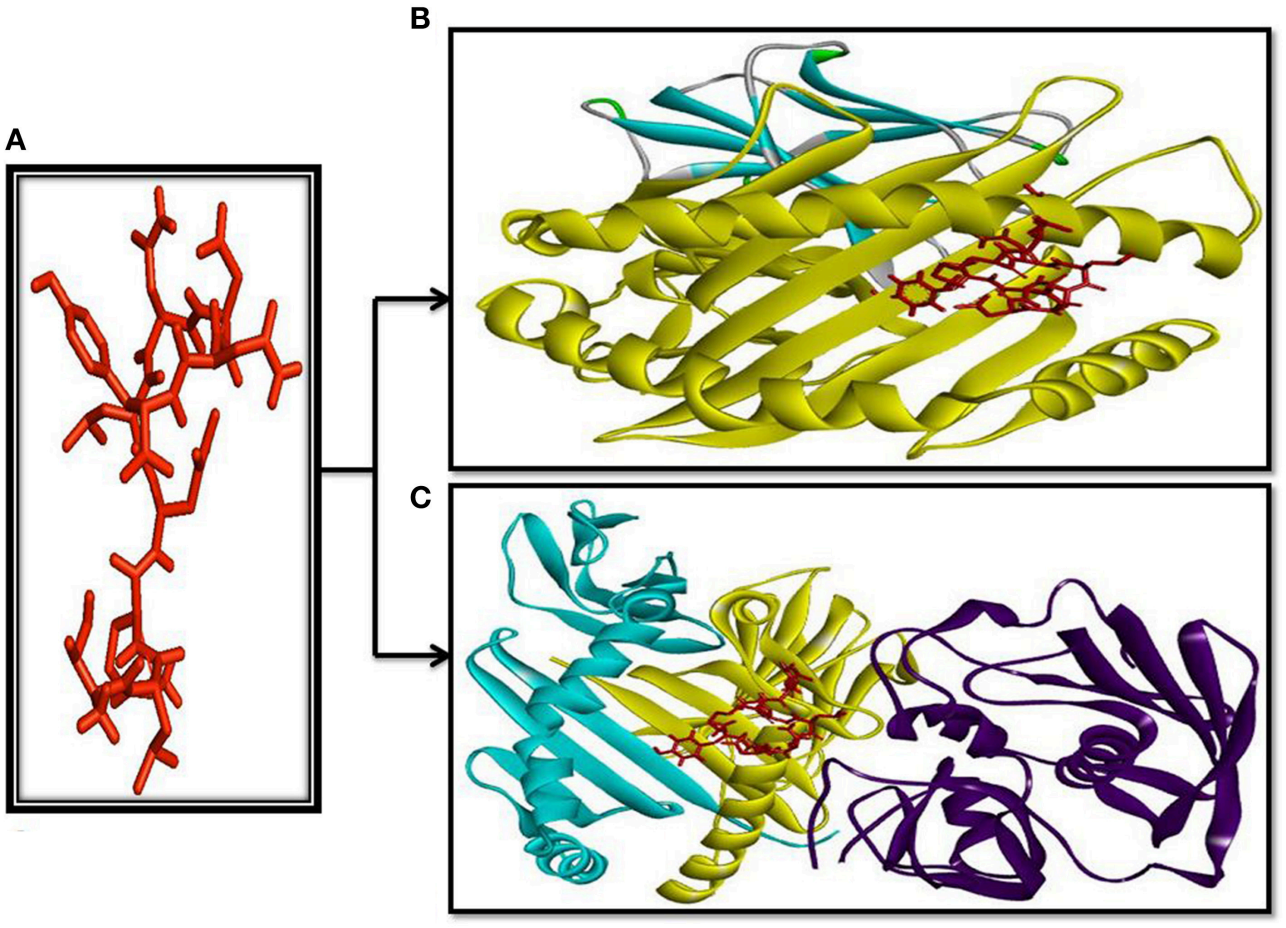
**Docking simulation study**. Structure of the predicted epitope *SU1*‘TLKNLNDNY’ (Red stick) **(A)** and visualize of docking results of *SU1* with MHC-I (HLAB^*^15:01); Chain A-yellow, Chain B-Bluish **(B)** as well as with MHC-II (HLA-DR) **(C)** Chain A-Yellow, Chain B-Bluish, Chain C-Blue.

AutoDockVina generated the binding modes of epitope TLKNLNDNY with HLA molecules. The interacted binding energy of the epitope TLKNLNDNY with HLA-B^*^15:01 receptor was known to be −7.4 and −7.0 kcal/mol for HLA-DR. The epitope 3D structure and its binding site to HLA are shown in Figure 6A (Epitope structure), Figure 6B (Epitope binds to MHC-I), and Figure 6C (Epitope binds to MHC-II).

### B cell epitope identification

**Figure 7:**
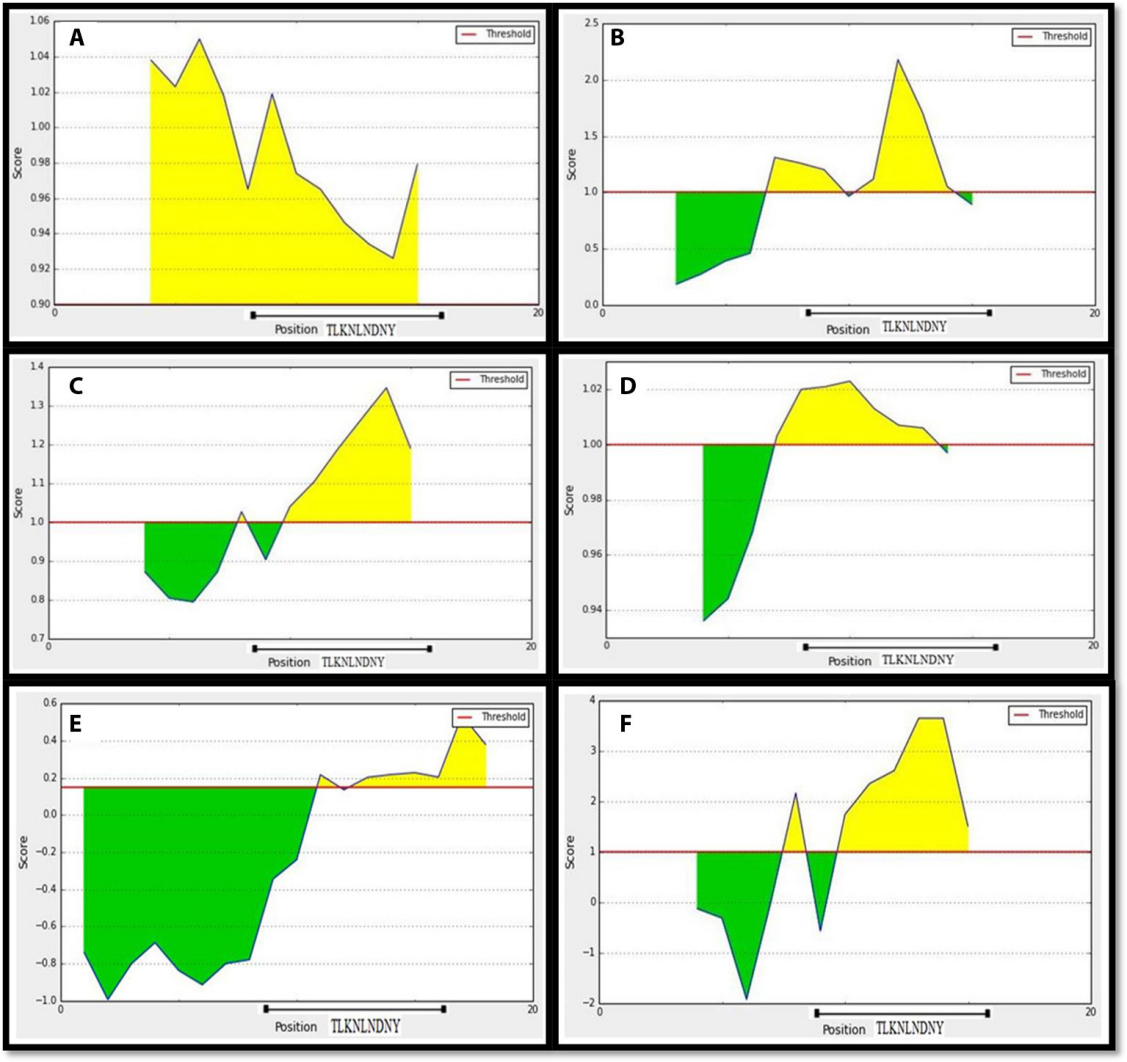
**Prediction of B-cell antigenic properties for most antigenic conserved region**. SU1 showed all the antigenic criteria to be predicted as B-cell epitope. **(A)** Kolaskar and Tongaonkar antigenicity prediction. **(B)** Emini surface accessibility prediction. **(C)** Chou and Fasman beta-turn prediction. **(D)** Karplus and Schulz flexibility prediction. **(E)** Bepipred linear epitope prediction. **(F)** Parker hydrophilicity prediction. The x-axis and y-axis represent the sequence position and corresponding antigenic properties score, respectively. The threshold level is 1.0 for most of the properties except for **(A)** (0.90) and **(E)** (0.19). The regions having antigenic properties are shown in yellow color above the threshold value.

**Figure 8:**
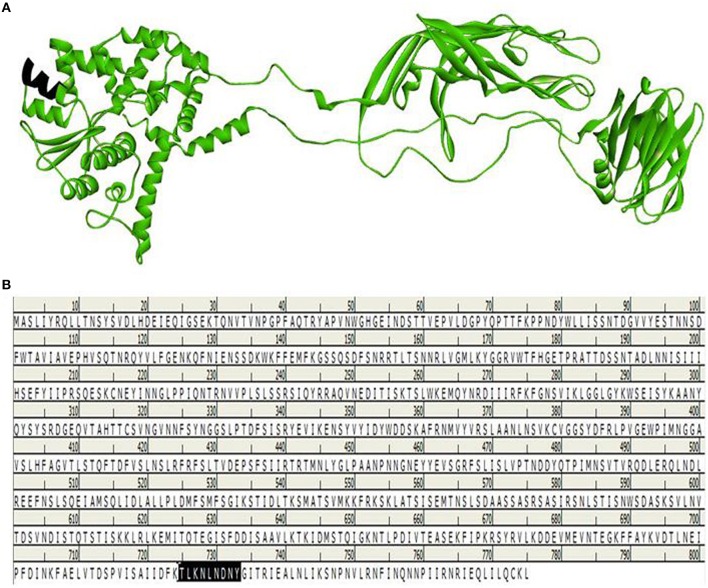
**Selected region for T-cell and B-cell epitope from built model**. Black region indicates the *SU1* epitope region of 3-D structure of VP4 protein **(A)**. Position of *SU1* epitope in linear amino acid sequence of VP4 protein **(B)**.

To recognize the potential B-cell epitopes, a number of analysis tools using amino scale based methodology were implemented on conserved region. The physio-chemical properties of amino acids from conserved region were evaluated by Kolaskar and Tongaonkar antigenicity tool that predicted an average antigenic propensity value of these amino acid as 1.058, with the maximum value of 1.240 and minimum of 0.920. The threshold for antigenic determination of conserved region was 1.00 whereas all values from these result >1.00 suggested as potential antigenic determinants. The region 720–731 from the conserved region meets the threshold value for the Kolaskar and Tongaonkar antigenicity property (Figure 7A). Amino acid residues 723–732 of the conserved region demonstrated as surface accessibility which is required to be an effective B cell epitope (Figure 7B). Beta turns of a protein or region can play significant role in prompting antigenicity as it can lead to hydrophilic properties and surface accessibility as well (Šali et al., 1995). The region 724–732 was considered as a β-turns region from Chou and Fasman Beta turn prediction (Figure 7C). It is speculated that the flexibility of the peptide have strong correlation with antigenicity (Söding, 2005). Karplus and Schulz flexibility prediction tool identified the region 723–731 to be the most flexible (Figure 7D). Bepipred linear epitope prediction tool was also employed in conserved region to identify the region which could be projected as linear B-cell epitope. And, in this case the region 726–735 was considered for linear epitope (Figure 7E). To confirm the hydrophilic nature of a peptide region parker hydrophilicity was also analyzed in the conserved region (Figure 7F). By cross-referencing all the data retrieved from all B-cell properties tools, we confirmed that the peptide sequence 725–733 from conserved regions capable of inducing an effective immune response as B cell epitope. The predicted T and B cell epitope was shown in Figure 8.

### Drug selection and its analog preparation

**Figure 9:**
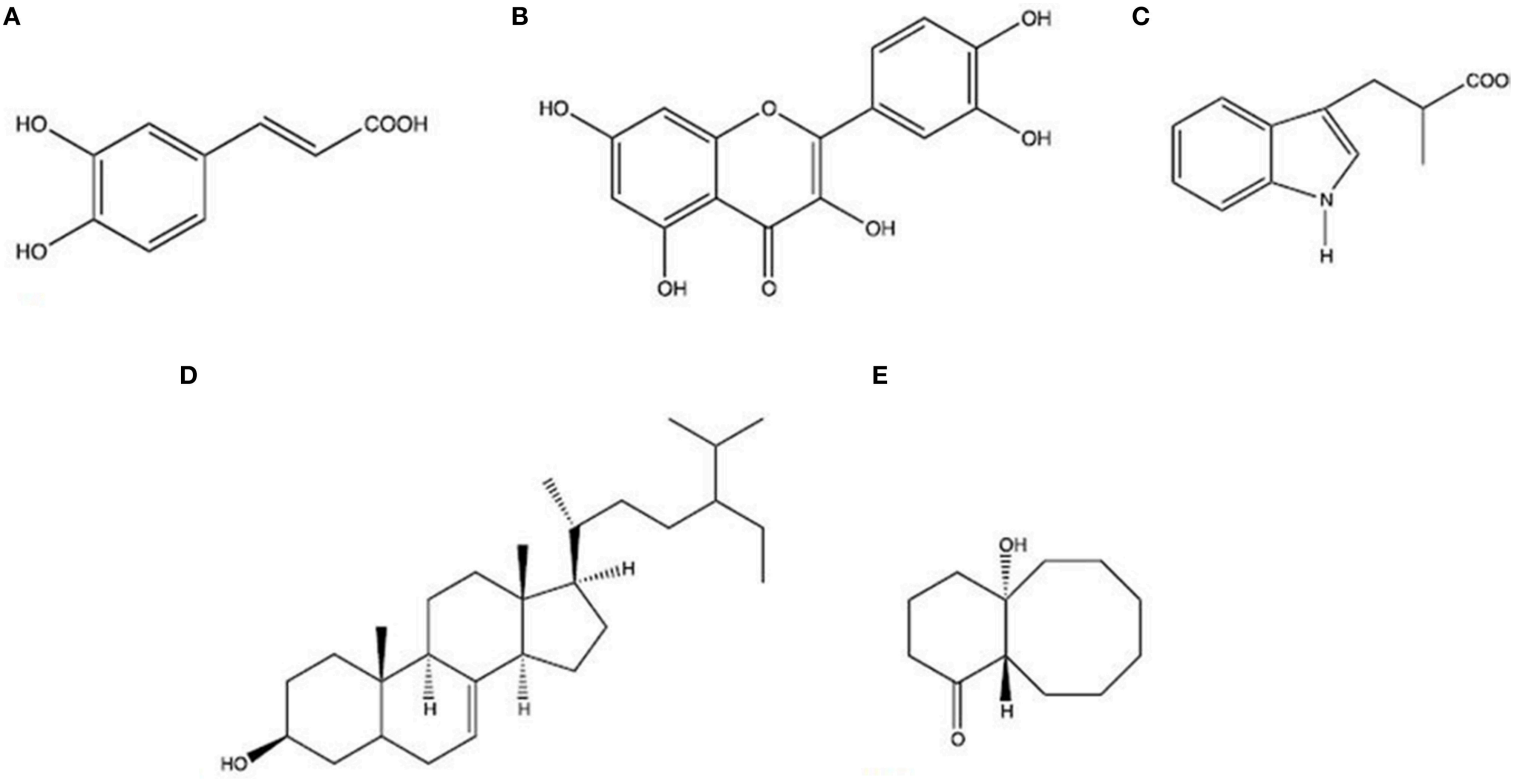
**Two dimensional (2D) structure of selected compounds from medicinal plant ***Rhizophora mucronata Lamk***. (A)** Caffeic acid, **(B)** Quercetin, **(C)** Rhizophorine, **(D)** Stigmast-7-en-3β-ol, **(E)** 1-Hydroxy-5 oxobicyclo[6.4.0] dodecane.

*Rhizophora mucronata Lamk. (Rhizophoraceae)* was selected for our study which is a mangrove's plant found in Sundarbans in Bangladesh and locally it is known as “Bhora.” Its size up to 15 m tall, with small white flowers and long ovoid-conical fruits (Wangensteen et al., 2013). This plant is also found in muddy shores and tidal creeks in tropical zones of East- and South Africa, Asia, Northeast Australia, and Central America (Kirtikar and Basu, 1935). The bark and leaves are traditionally used in diarrheal patient. Quercetin and caffeic acid, isolated compounds from bark, appeared to have the highest activity as antidiarrheal (Rohini and Das, 2010). Whereas isolated compounds, Stigmast-7-en-3β-ol, Rhizophorine, 1-Hydroxy-5-oxobicyclo [6.4.0] dodecane, with methanol extracts from leaves were also demonstrated as antidiarrheal (Puspitasar et al., 2012). The 2D structure of these molecules were depicted in Figure 9.

### Energy minimization analysis

YASARA program was used in refining physical realism, stereochemistry, and side-chain accurateness in homology modeling and designed molecules. This program yields the energy (START vs. END) that ensures accuracy of modeled protein and designed molecules (Figure S5). The End energy of homology modeling was −383,578.5 kJ/mol compared to START energy −101,952.5 kJ/mol, revealed that the model protein is more accurate than the prior modeled protein. In the case of drawn molecules the energy comparison from the START energy Rhizophorine (12,454.4 kJ/mol) and 1-Hydroxy-5 oxobicyclo[6.4.0]dodecane (4473.9 kJ/mol) to END energy is Rhizophorine (471.5 kJ/mol) and 1-Hydroxy-5 oxobicyclo[6.4.0]dodecane (210.2 KJ/mol) (Figure S5).

### Docking and active site analysis

**Figure 10:**
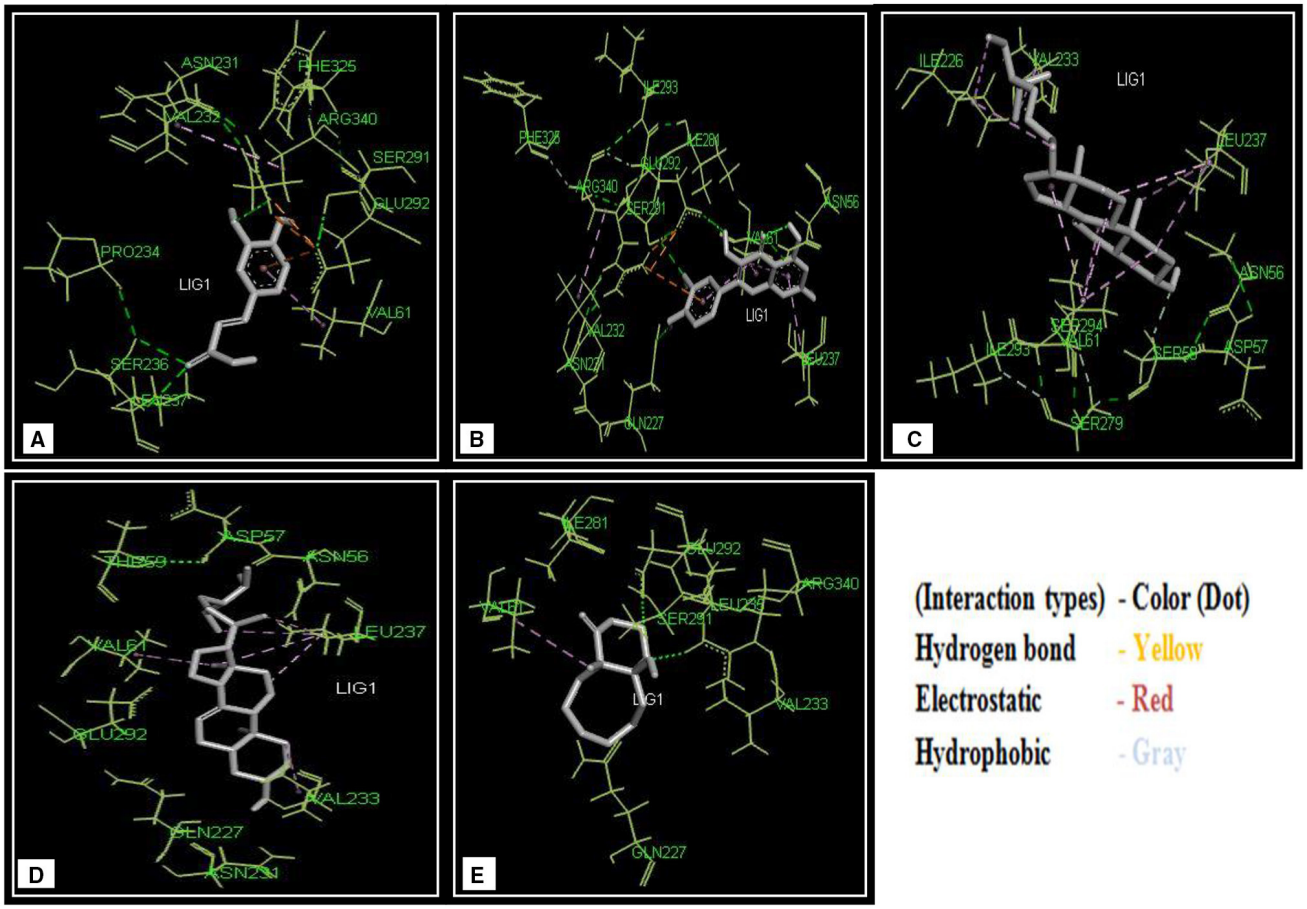
**Drug compounds binding amino acid residues of VP4 3D model. (A)** Caffeic acid, **(B)** Quercetin, **(C)** Rhizophorine E, **(D)** Stigmast-7-en-3β-ol, **(E)** 1-Hydroxy-5-oxobicyclo [6.4.0] dodecane.

**Figure 11:**
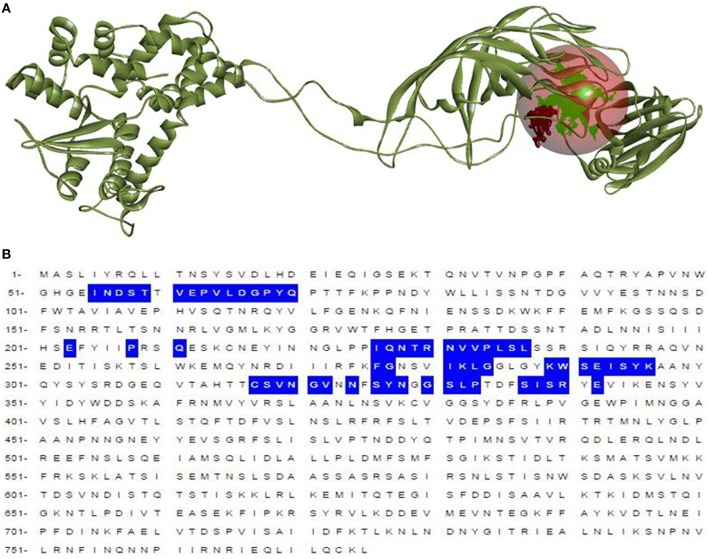
**Interactions of drug compounds with the VP4 protein. (A)** Binding site of drug compounds in VP4 protein. Circled area depicts the binding site of VP4 protein. **(B)** Active site residues (Blue) of VP4 protein model.

The two most convenient docking tools Autodock 4.2 and AutodockVina of MGL 1.5.6 were employed to analyze the protein–drug interactions. All docking run was performed between the modeled protein and drug compounds with the set of docking parameters mentioned in Materials and Methods Section. The docking energy of these drug molecules in AutoDockVina (Caffeic acid: −6.7 kJ/mol, Quercetin: −8.3 kJ/mol, Stigmast-7-en-3β-ol: −7.24 kJ/mol, Rhizophorine: −7.7 kJ/mol, 1-Hydroxy-5-oxobicyclo [6.4.0] dodecane: −8.0 kJ/mol) and AutoDock 4.2 (Caffeic acid: −5.99 kJ/mol, Quercetin: −7.24 kJ/mol, Stigmast-7-en-3β-ol: −6.67 kJ/mol, Rhizophorine: −6.88 kJ/mol, 1-Hydroxy-5-oxobicyclo [6.4.0] dodecane: −7.3 kJ/mol) tabulated in Table 5 which suggest the higher binding affinity. These analyses showed that the drug molecules bind into the binding site shown in Figure 10. The analysis of binding site also revealed that the interacting residues bind into the binding site shown in Figure 11A. And all the residues are the active residues of binding site shown in Figure 11B. It was interesting to observe that all drug compounds which were analyzed for this study interacted with the most common three residues (Val-61, Leu-237, Glu-292) shown in Table 5.

**Table 5:** **Docking result of medicinal plant compounds**.

**Inhibitors**	**Residues name**	**Docking energy/binding affinity (Kcal/mol)**		**Inhibitor constant Ki (μm)**	**Ligand efficieny**	**Intermolecular energy**	**Electrostatic energy**
		**AutodockVina**	**Autodock 4.2**				
Caffeic acid	Val-61, Asn-231, Val-232, Pro-234, Ser-236, Leu-237, Ser-291, Glu-292, Phe-325, Arg-340	−6.7	−5.99	40.44	0.92	−6.92	−1.65
Quercetin	Asn-56, Val-61, Glu-227, Asn-231, Val-232, Pro-234, Ser-236, Leu-237, Ile-281, Ser-291, Glu-292, Ile-293, Phe-325, Arg-340	−8.3	−7.42	63.87	0.9	−7.17	−1.22
Stigmast-7-en-3β-ol	Asn-56, Asp-57, ser-58, Val-61, Ile-226, Val-233, Leu-237, Ser-279, Ile-293, Ser-294	−7.24	−6.67	49.33	0.7	−5.99	−2.43
Rhizophorine	Asn-56, Asp-57, Thr-59, Val-61, Glu-227, Asn-231, Val-233, Ile-237, Glu-292	−7.7	−6.88	27.48	0.55	−6.33	−2.34
1-Hydroxy-5-oxobicyclo [6.4.0]dodecane	Val-61, Glu-227, Val-233, Leu-235, Ile-281, Ser-291, Glu-292, Arg-340	−8.0	−7.3	51.48	0.63	−5.49	−1.54

### QSAR and pharmacoinformatics analysis

**Figure 12:**
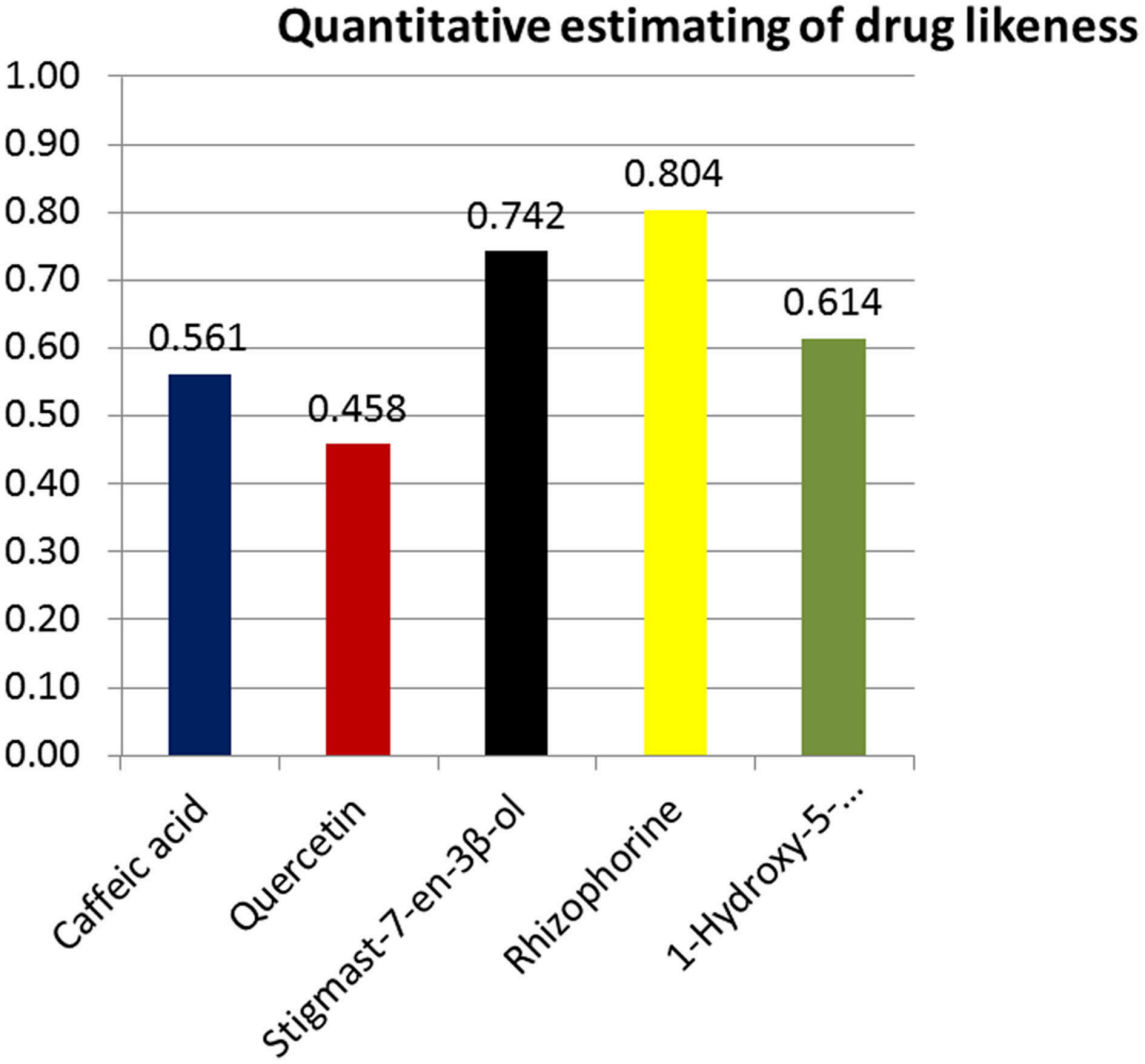
**Quantitative estimation of drug likeness**. Rhizophorine showed the highest score of drug likeness (0.804) compared to other compounds.

**Figure 13:**
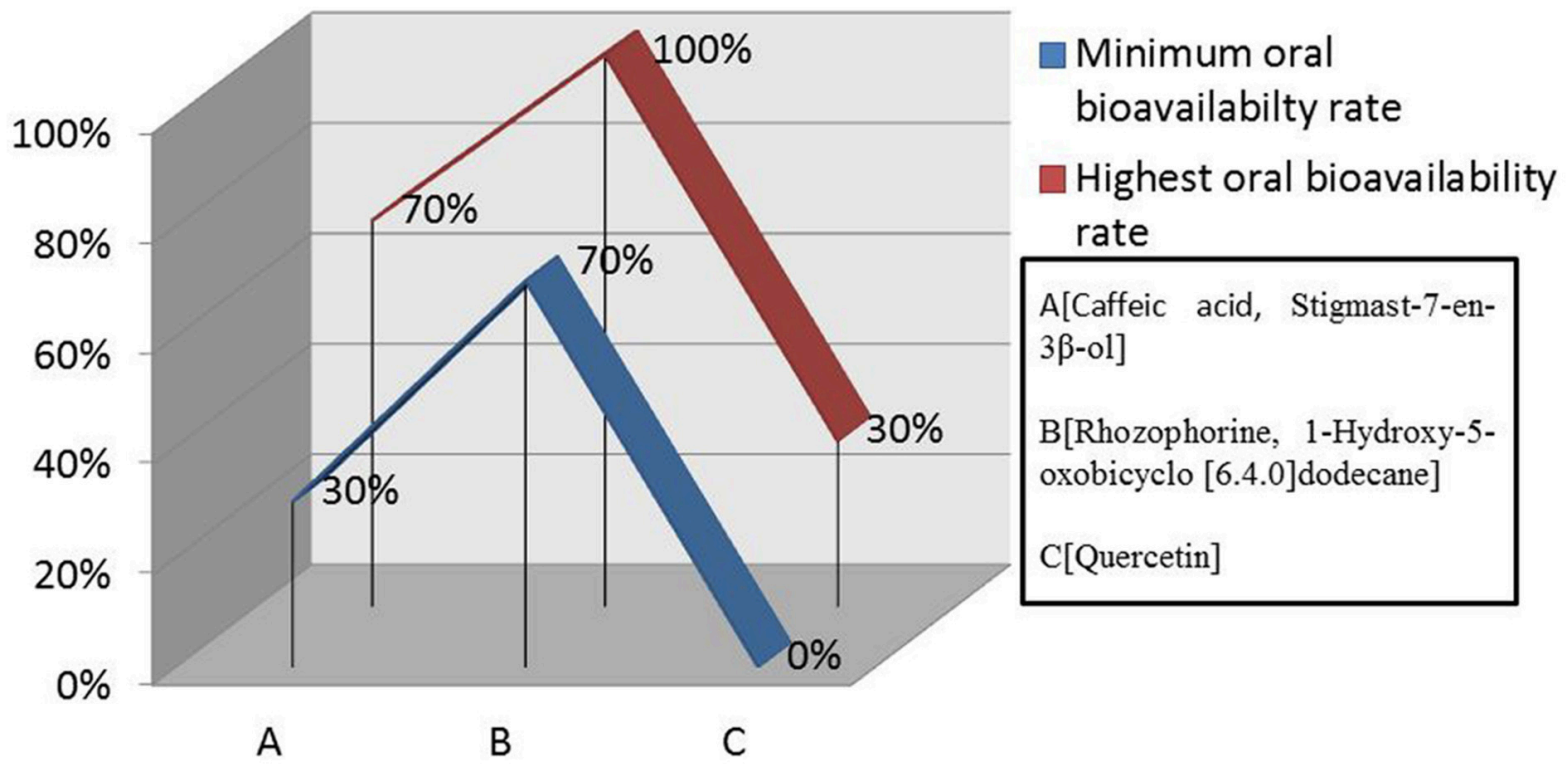
**Oral bioavailability rate of compounds**. Rhizophorine and 1-Hydroxy-5-oxobicyclo [6.4.0] dodecane indicated higher oral bioavailability rate (70–100%) than the other plant medicinal drug compounds.

The outcomes from OSIRIS property explorer, ACToR and Molinspiration have been tabulated to analyze the compounds quantitative structural activity relationship and structural polarity (Table 6) which confirm them as possible drug candidates. In this study we also verified the overall toxicity and ADME (Absorption, Distribution, metabolism, Excretion) using admetSAR, ACD/I-lab, AcTor and Osiris program. admetSAR and ACD/I-lab prediction methods involves Human Intestinal Absorption (HIA), Skin permeability, Blood-Brain Barrier distribution (LogBB), Caco-2 cell permeability, Volume of distribution, CYP450 2C9 substrate, and inhibitors. The Osiris program was used for the generation of toxicity profile of selected compounds. The result of these studies is given in Table 7. The DruLiTo software suggests that the medicinal plant drug compounds have higher drug likeness to be expected as antidiarrheal drug. The drug likeness of Caffeic acid (0.561), Quercetin (0.458), Stigmast-7-en-3β-ol(0.742), Rhizophorine(0.804), 1-Hydroxy-5 oxobicyclo[6.4.0] dodecane(0.614) (Figure 12) as well as oral bioavailabilty (Figure 13 and Table 6) confirm their drug efficacy. Besides these drug compounds also confirmed that there are no mutagenic, irritative, reproductive toxicity, and carcinogenic profile.

**Table 6:** **QSAR properties of medicinal plant compounds**.

**Ligand properties**	**Caffeic acid**	**Quercetin**	**Stigmast-7-en-3β-ol**	**Rhizophorine**	**1-Hydroxy-5-oxobicyclo [6.4.0]dodecane**
Molecular weight(g/mol)	180.16	302.24	414.72	203.24	210.32
No. of H donor	3	5	1	2	1
No. of H acceptor	4	7	1	3	2
No. of rotatable bonds	2	1	6	3	0
No. of hydrogen bond	7	12	2	5	3
logP	0.888	1.834	11.785	1.265	−1.568
TPSA	77.75	131.35	20.23	53.09	37.30
unwQED	0.655	0.467	0.617	0.841	0.624
Oral bioavailability	Between 30 and 70%	< 30%	Between 30 and 70%	More than 70%	More than 70%

**Table 7:** **ADMET properties of the medicinal plant compounds**.

**Properties**	**Caffeic acid**	**Quercetin**	**Stigmast-7-en-3β-ol**	**Rhizophorine**	**1-Hydroxy-5-oxobicyclo [6.4.0]dodecane**
**ABSORPTION**					
Renal organic cation transporter	0.9387	0.9310	0.7866	0.9131	0.8457
Blood brain barrier	0.6322	0.5711	0.9820	0.9847	0.9692
Human intestinal absorption	0.9392	0.9650	1.0000	1.0000	1.0000
Caco-2 permeability	0.5693	0.8957	0.8147	0.5403	0.8182
**DISTRIBUTION**					
DBP (%PPB)	46.57%	93.43%	99.52%	92.82%	52.94%
Blood brain distribution (logBB)	−0.16	0.53%	−0.02	−0.74	0.44
Volume of distribution (Vd)	0.31 L/k	0.6 L/kg	6.27 L/kg	0.31 L/kg	1.53 L/kg
**METABOLISM**					
CYP450 2C9 substrate	0.8014	0.7898	0.8219	0.7614	0.7915
CYP450 2C9 inhibitor	0.9071	0.5823	0.8613	0.8025	0.9021
**TOXICITY**					
Violation	No	No	No	No	No
Mutagenecity	No	No	No	No	No
Tumorogenecity	No	No	No	No	No
Irritating effects	No	No	No	No	No
Reproductive effects	No	No	No	No	No

## Discussion

Now a days, developing country is being more sufferer as the new diseases and severity of diseases are evolving along with new viruses in parallel to advancement of the medical technology. However, medical science has always tried to cope up with the problems of replicating disease. Human rotavirus infection is one of the reasons of fatalities in humans in a number of countries like Bangladesh, India, Thailand, USA, Australia, UK, etc. To cure this type of diseases both vaccines and drugs are compulsory. However, only wet lab approach couldn't allow the faster method for drug and vaccine design.

The advancement of new vaccine is very important to resist this deadly virus. Recently, bioinformatics tools have been proved very effective to design peptide based vaccine to target a specific protein. Epitope-based vaccine designing against rhinovirus (Lapelosa et al., 2009) and dengue virus (Chakraborty et al., 2010) have already been recommended. In spite of being an acquainted concept, epitope-based vaccine designing hasn't yet been utilized against VP4 protein. Although vaccine designing based on B cell immunity is a common concept, but both T cell and B cell epitope are encouraged now a days as both of them produce strong immune response (Shrestha and Diamond, 2004). In this study, we have used different bioinformatics tools to predict both B and T cell epitopes to confer better immunity in rotavirus.

To develop an epitope based vaccine against rotavirus effective for the globe we have analyzed the distance among 24 available alleles of VP4 protein available from 14 different countries. As a consequence of small distances among the allele, four conserved regions could be identified among the 24 VP4 protein alleles (Figures 2, 3, Table 1 and Figure S1). The conserved region showing the highest antigenicity (antigenic score 0.9606) was considered as the potent region (SAIIDFKTLKNLNDNYGI) of epitope prediction (Table 1). Among the predicted most potent five T cell epitopes derived from conserved region 4, peptide region 725–733 “TLKNLNDNY” termed as *SU1* showed the highest NetCTL score (1.048) as well as the highest conservancy rate 65.66% (Table 2). *SU1* epitope is expected to provide the protection broadly as this epitope is conserved against all the available rotavirus strains. The specific high affinity binding of *SU1* with 11 HLA-I as well as HLA DR and DQ molecules is anticipated as the efficiency of this epitope vaccine's precise interaction with HLA alleles. It is to be noted that more interactions with MHC-1 molecules were observed when higher concentrations (>500 nM) were considered (data not shown; Tables 3, 4). To confirm the binding affinity, *SU1* epitope was docked to both MHC-I and MHC-II allele HLA-B^*^15:01 and HLA-DR, respectively (Figure 6). The docking analysis confirmed the *SU1* epitope binding with the MHC-alleles. The selected alleles responsible for higher affinity by *SU1* were found to be covered for at least half of the population of the corresponding 14 countries (Figure 5). We have also searched for B cell epitopes in the conserved region as B cell epitopes can induce both primary and secondary immunity. All the six set of tools also confirmed the presence of antigenicity, beta turns, linearity, flexibility, accessibility and hydrophillicity of *SU1* as a potent B-cell epitope. According to these results that the vaccine would be effective for a vast population throughout a wide geographical region.

The chronic stage of the rotavirus infection can last for long times. Therefore, for minimization or complete elimination of chronic symptoms caused by the infection, a therapeutic agent is required. Furthermore, a universal drug is essential to completely eradicate the rotavirus infection. If mutation in the virus causes reduction of the effectiveness of the vaccine, the drug may work alongside to diminish the symptoms faster. In the worst case, sudden rotavirus outbreak in a non-vaccinated area vaccination might become outdated. Only post-therapeutic measure or drug would be effective until new vaccine kicks in. In the present study along with pre therapy vaccine development we also predicted the active sites on the highest antigenic protein of the rotavirus and subsequently predicted the novel inhibitors of the protein to generate post therapy.

To have the better therapeutic strategies against rotavirus induced diarrhea some novel medicinal drug compounds (Figure 9) were analyzed by bioinformatics analysis. Experimental data suggest that the plant medicinal compounds have considerable values of antidiarrheal activities but their exact modes of action yet to be investigated. Drug design studies focus on drug-protein interactions and these interactions are usually considered effective when found in active pocket of binding site of the protein (Hossain et al., 2016). According to this hypothesis, modeled VP4 protein and selected plant compounds were investigated for their appropriate binding. With two most suitable docking programs, we have identified their interacting residues as Val-61, Leu-237, and Glu-292. The ligand–receptor interactions Val-61 were found in all the drug compounds. All of these interacting amino acids are placed in the active pocket of the VP4 protein (Figures 10, 11). The drug nature of the medicinal compounds were confirmed by acceptable QSAR (Quantitative structure activity relationship) and ADMET/Tox properties (Tables 6, 7). Among the ADMET properties, Stigmast-7-en-3β-ol, Rhizophorine and 1-Hydroxy-5-oxobicyclo [6.4.0] dodecane show the same human intestinal absorption rate (1.000) than the Caffeic acid (0.9392) and Quercetin (0.9650). The drugs effectiveness also assured as no signs to cross Blood Brain barrier observed. As expected, blood brain distribution of the drug compounds are less than zero except Quercetin (0.53). Volume of distribution of drugs is quite good into body as the score is ranging from 0.31 to 6.27 l/kg. The satisfactory metabolic score (0.5823–0.9071) was perceived for five drug compounds. And no toxic effect would be expected from these compounds treated to the rotaviral mediated diarrheal patient.

Also the higher drug likeness (Figure 12) and oral bioavailability (Figure 13) of all medicinal drug compounds support their effectiveness as antidiarrheal drugs. Thus, these molecules could be possible inhibitory agents of VP4 protein against rotavirus A.

However, experimental validations for these predicted epitopes are indeed necessary for their practical application. Experiments for synthesized peptides might include T-cell proliferation and flowcytometry for activated B-cell with specific markers (Geluk et al., 2000, 2009; De Groot et al., 2001; McMurry et al., 2007; Davila et al., 2012; Commandeur et al., 2014; Khan et al., 2014). For the drug discovery, the suggested drugs have to be synthesized to test the efficacy in animal model experiment. Thereafter, both preclinical and clinical study should be done before the drug marketing.

Finally, after experimental validation the suggested *SU1* peptide for T and B-cell epitope may efficiently be used for the improvement of peptide vaccine to provoke an absolute immune response against VP4 protein of rotavirus. Moreover, identification of novel plant medicinal compounds are expected to be successful inhibitor against the rotavirus.

## Conclusion

We have identified a potential peptide region from VP4 protein of human rotavirus A for vaccine design which is likely to prompt both T and B-cell mediated immune responses. The study was performed by utilizing computational approaches based on sequence conservancy, antigenicity and its allelic interactions. In addition, we have proposed compounds from medicinal plant *Rhizophora mucronata Lamk*. which might be effective against rotavirus A as antidiarrheal drugs. Absence of accurate investigational disease model, however, can hinder to confirm this *in silico* study for rotavirus. So it is therefore to be needed suitable animal model experiments for the validation of the proposed epitopes and drugs.

## Author contributions

MS: Conceived, designed, and guided the study, analyzed the data, helped in drafting and performed critical revision. CAK: Guided the study, acquisition and analyzed the data, helped in drafting the manuscript. AH: Analyzed the data and helped in drafting the manuscript. MUH: Helped to design the study, performed bioinformatics analysis, drafted and developed the manuscript, and performed critical revision. All authors have approved the manuscript.

### Conflict of interest statement

The authors declare that the research was conducted in the absence of any commercial or financial relationships that could be construed as a potential conflict of interest. The authors declare that they have competing interests on proposed vaccines and drugs.

## Abbreviations

CADD: Computer Aided Drug Design
Con.: Conduritol
3D: three dimensional
PDB: Protein data bank
ADME/Tox: Absorption, Distribution, Metabolism, Excretion, Toxicity
LogS: Logarithm of solubility
cLogP: Logarithm of partition coefficient
TPSA: The polar surface area.
QSAR: Quantitative structural activity relationship
HIA: Human Intestinal Absorption
Vd: Volume of Distribution
UniPortKB: UniProt Knowledge Base
CTL: Cytotoxic T-lymphocyte
IC50: half-maximal Inhibitory Concentration
MHC: Major Histocompatibility Complex
IEDB: Immune Epitope Database
TAP: Transport Associated Proteins
ACToR: Aggregated Computational Toxicology Resource
admetSAR: absorption, distribution, metabolism, excretion and toxicity Structure-Activity Relationship database
nM: Nanomolar
nm: Nanometer
MSA: Multiple Sequence Alignment.
