# Supplementary material for: Therapeutics Insight with Inclusive Immunopharmacology Explication of Human Rotavirus A for the Treatment of Diarrhea
Source: Front Pharmacol. 2016 Jun 23;7:153. doi: 10.3389/fphar.2016.00153 (PMC4917548; doi:10.3389/fphar.2016.00153)
Supplement: Supplementary file 1 [file Table1.PDF]

S1 Table: Node type (Root, Internal node and Leaf), Branch length and Bootstrap values (Internal Node) of 24 strains of rotavirus.

Strain Name	Node Type	Branch length	Bootstrap Value
sp|B3SRX5| United States	Leaf	4.49E-3	Non-applicable
	Internal Node	4.49E-3	Non-applicable
	Internal Node	1.37E-3	48
	Internal Node	0.01	100
	Internal Node	3.41E-3	52
	Internal Node	0.02	98
	Internal Node	0.03	100
	Internal Node	4.73E-3	88
gi|320117675|gb|ADW11134.1| Bangladesh	Leaf	0.01	Non-applicable
sp|P21284| Philippines	Leaf	0.02	Non-applicable
	Internal Node	5.99E-3	76
sp|P30214| Australia	Leaf	0.01	Non-applicable
sp|P11196|United States	Leaf	0.01	Non-applicable
	Internal Node	0.07	100
	Internal Node	0.02	70
	Internal Node	0.04	86
	Internal Node	0.02	92
	Internal Node	0.05	100
sp|Q06895|Israel	Leaf	0.09	Non-applicable
sp|P26451|Indonesia	Leaf	0.08	Non-applicable
	Internal Node	0.15	100
	Internal Node	0.04	100
	Internal Node	9.77E-3	92
sp|B3SRR1|United Kingdom	Leaf	0.03	Non-applicable
sp|Q08778|Thailand	Leaf	0.03	Non-applicable
sp|Q3ZK58|Belgium	Leaf	0.04	Non-applicable
	Internal Node	0.07	100
sp|Q01641|Japan	Leaf	0.01	Non-applicable
sp|P39033|Japan	Leaf	5.49E-3	Non-applicable
sp|Q09113|India	Leaf	0.36	Non-applicable
sp|P11199| Australia	Leaf	0.01	Non-applicable
sp|P11198|Sweden	Leaf	0.02	Non-applicable
sp|P11200|United kingdom	Leaf	0.02	Non-applicable
sp|P11197|Venezuella	Leaf	0.02	Non-applicable
	Internal Node	0.02	100
	Internal Node	3.74E-3	99
sp|BS3RR9|United States	Leaf	0.02	Non-applicable
sp|P11193|United states	Leaf	0.02	Non-applicable
sp|P11194|Italy	Leaf	0.03	Non-applicable

sp|P11195|United states	Leaf	0.03	Non-applicable
sp|Q9WAK4|Japan	Leaf	0.03	Non-applicable
	Internal Node	2.13E-3	62
sp|Q9WAK4|Japan	Leaf	0.02	Non-applicable
sp|P13842|Japan	Leaf	5.93E-3	Non-applicable
