# Supplementary material for: Therapeutics Insight with Inclusive Immunopharmacology Explication of Human Rotavirus A for the Treatment of Diarrhea
Source: Front Pharmacol. 2016 Jun 23;7:153. doi: 10.3389/fphar.2016.00153 (PMC4917548; doi:10.3389/fphar.2016.00153)
Supplement: Supplementary file 2 [file Image1.PDF]

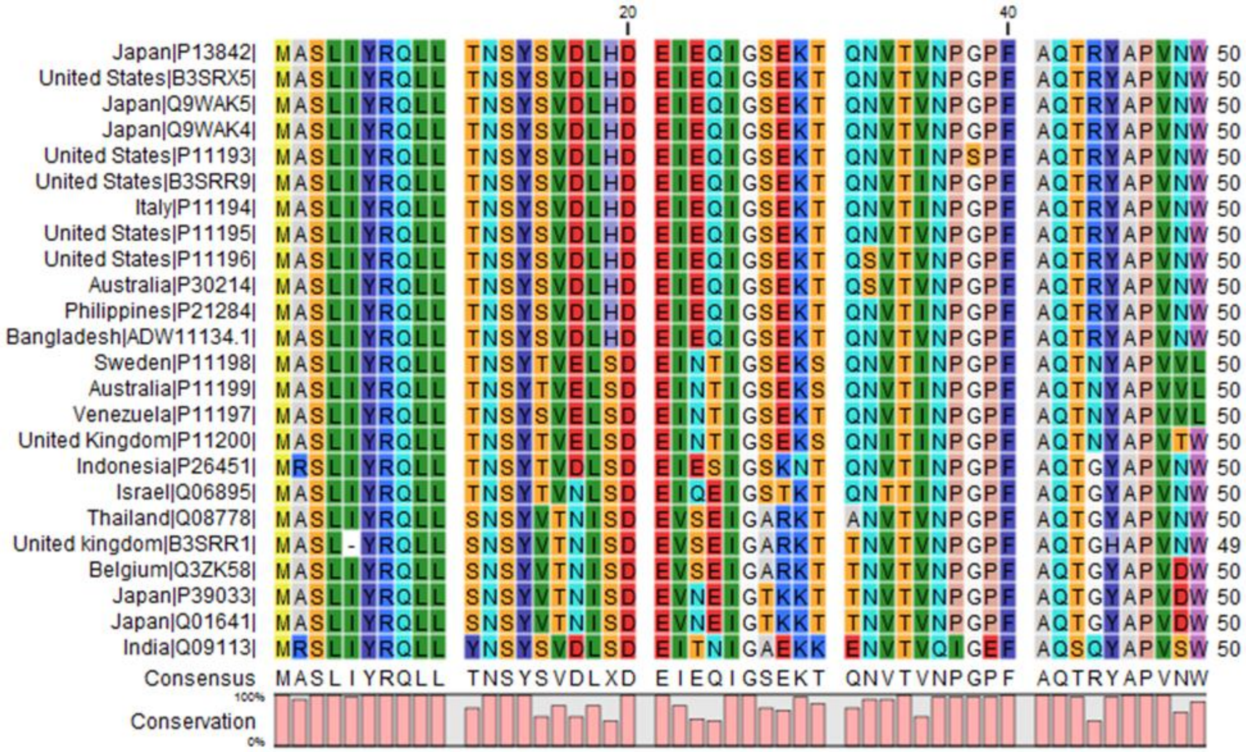
20
40
Japan|P13842| MASLIYRQLL TNSYSVDLHD EIEQIGSEKT QNVTVNPGPF AQTRYAPVNW 50
United States|B3SRX5| MASLIYRQLL TNSYSVDLHD EIEQIGSEKT QNVTVNPGPF AQTRYAPVNW 50
Japan|Q9WAK5| MASLIYRQLL TNSYSVDLHD EIEQIGSEKT QNVTVNPGPF AQTRYAPVNW 50
Japan|Q9WAK4| MASLIYRQLL TNSYSVDLHD EIEQIGSEKT QNVTVNPGPF AQTRYAPVNW 50
United States|P11193| MASLIYRQLL TNSYSVDLHD EIEQIGSEKT QNVTINPSPF AQTRYAPVNW 50
United States|B3SRR9| MASLIYRQLL TNSYSVDLHD EIEQIGSEKT QNVTINPGPF AQTRYAPVNW 50
Italy|P11194| MASLIYRQLL TNSYSVDLHD EIEQIGSEKT QNVTINPGPF AQTRYAPVNW 50
United States|P11195| MASLIYRQLL TNSYSVDLHD EIEQIGSEKT QNVTVNPGPF AQTRYAPVNW 50
United States|P11196| MASLIYRQLL TNSYSVDLHD EIEQIGSEKT QSVTVNPGPF AQTRYAPVNW 50
Australia|P30214| MASLIYRQLL TNSYSVDLHD EIEQIGSEKT QSVTVNPGPF AQTRYAPVNW 50
Philippines|P21284| MASLIYRQLL TNSYSVDLHD EIEQIGSEKT QNVTINPGPF AQTRYAPVNW 50
Bangladesh|ADW11134.1| MASLIYRQLL TNSYSVDLHD EIEQIGSEKT QNVTVNPGPF AQTRYAPVNW 50
Sweden|P11198| MASLIYRQLL TNSYTVELSD EINTIGSEKS QNVTINPGPF AQTNYAPVVL 50
Australia|P11199| MASLIYRQLL TNSYTVELSD EINTIGSEKS QNVTINPGPF AQTNYAPVVL 50
Venezuela|P11197| MASLIYRQLL TNSYSVELSD EINTIGSEKT QNVTINPGPF AQTNYAPVVL 50
United Kingdom|P11200| MASLIYRQLL TNSYTVELSD EINTIGSEKS QNITINPGPF AQTNYAPVTW 50
Indonesia|P26451| MRSLIYRQLL TNSYTVDLSD EIESIGSKNT QNVTINPGPF AQTGYAPVNW 50
Israel|Q06895| MASLIYRQLL TNSYTVNLSD EIQEIGSTKT QNTTINPGPF AQTGYAPVNW 50
Thailand|Q08778| MASLIYRQLL SNSYVTNISD EVSEIGARKT ANVTVNPGPF AQTGYAPVNW 50
United kingdom|B3SRR1| MASL-YRQLL SNSYVTNISD EVSEIGARKT TNVTVNPGPF AQTGHAPVNW 49
Belgium|Q3ZK58| MASLIYRQLL SNSYVTNISD EVSEIGARKT TNVTVNPGPF AQTGYAPVDW 50
Japan|P39033| MASLIYRQLL SNSYVTNISD EVNEIGTKKT TNVTVNPGPF AQTGYAPVDW 50
Japan|Q01641| MASLIYRQLL SNSYVTNISD EVNEIGTKKT TNVTVNPGPF AQTGYAPVDW 50
India|Q09113| MRSLIYRQLL YNSYSVDLSD EITNIGAEKK ENVTVQIGEF AQSQYAPVSW 50
Consensus MASLIYRQLL TNSYSVDLXD EIEQIGSEKT QNVTVNPGPF AQTRYAPVNW
100%
Conservation
0%

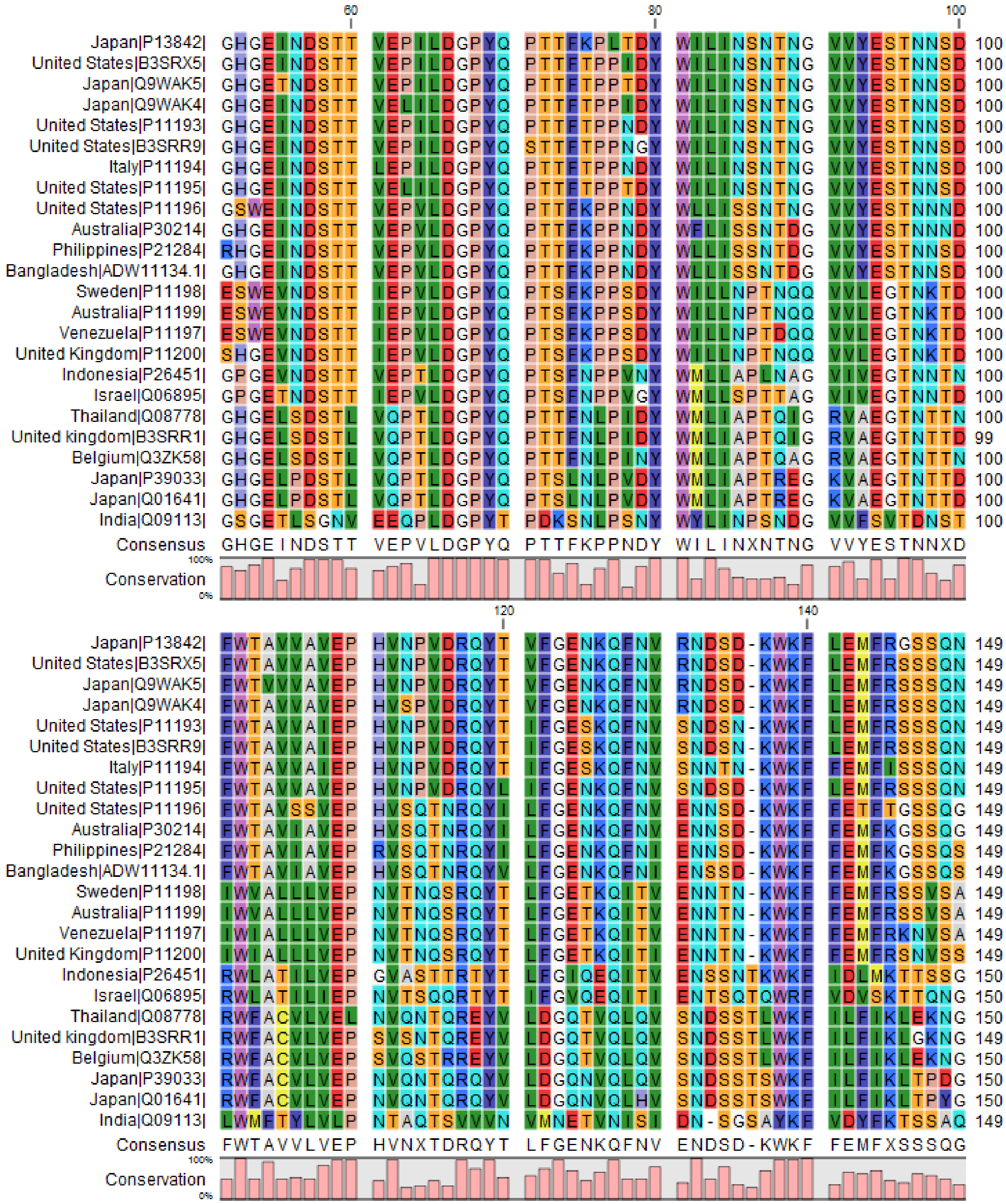
60
80
100
Japan|P13842| GHGEINDSTT VEPILDGPYQ PTTFKPLTDY WILINSNTNG VVYESTNNSD 100
United States|B3SRX5| GHGEINDSTT VEPILDGPYQ PTTFTPPIDY WILINSNTNG VVYESTNNSD 100
Japan|Q9WAK5| GHGETNDSTT VEPILDGPYQ PTTFTPPTDY WILINSNTNG VVYESTNNSD 100
Japan|Q9WAK4| GHGEINDSTT VELILDGPYQ PTTFTPPIDY WILINSNTNG VVYESTNNSD 100
United States|P11193| GHGEINDSTT VEPILDGPYQ PTTFTPPNDY WILINSNTNG VVYESTNNSD 100
United States|B3SRR9| GHGEINDSTT VEPILDGPYQ STTFTPPNGY WILINSNTNG VVYESTNNSD 100
Italy|P11194| GHGEINDSTT LEPILDGPYQ PTTFTPPNDY WILINSNTNG VVYESTNNSD 100
United States|P11195| GHGEINDSTT VELILDGPYQ PTTFTPPTDY WILINSNTNG VVYESTNNSD 100
United States|P11196| GSWEINDSTT VEPVLDGPYQ PTTFKPPNDY WLLISSNTNG VVYESTNNND 100
Australia|P30214| GHGEINDSTT VEPVLDGPYQ PTTFKPPNDY WFLISSNTDG VVYESTNNND 100
Philippines|P21284| RHGEINDSTT VEPVLDGPYQ PTTFKPPNDY WLLISSNTDG VVYESTNNSD 100
Bangladesh|ADW11134.1| GHGEINDSTT VEPVLDGPYQ PTTFKPPNDY WLLISSNTDG VVYESTNNSD 100
Sweden|P11198| ESWEVNDSTT IEPVLDGPYQ PTSFKPPSDY WILLNPTNQQ VVLEGTNKTD 100
Australia|P11199| ESWEVNDSTT IEPVLDGPYQ PTSFKPPSDY WILLNPTNQQ VVLEGTNKTD 100
Venezuela|P11197| ESWEVNDSTT IEPVLDGPYQ PTSFKPPSDY WILLNPTDQQ VVLEGTNKTD 100
United Kingdom|P11200| SHGEVNDSTT IEPVLDGPYQ PTSFKPPSDY WILLNPTNQQ VVLEGTNKTD 100
Indonesia|P26451| GPGEVNDSTT VEPTLDGPYQ PTSFNPPVNY WMLLAPLNAG VIVEGTNNTN 100
Israel|Q06895| GPGETNDSTT IEPVLDGPYQ PTSFNPPVGY WMLLSPTTAG VIVEGTNNTD 100
Thailand|Q08778| GHGELSDSTL VQPTLDGPYQ PTTFNLPIDY WMLIAPTQIG RVAEGTNTTN 100
United kingdom|B3SRR1| GHGELSDSTL VQPTLDGPYQ PTTFNLPIDY WMLIAPTQIG RVAEGTNTTD 99
Belgium|Q3ZK58| GHGELSDSTL VQPTLDGPYQ PTTFNLPINY WMLIAPTQAG RVAEGTNTTN 100
Japan|P39033| GHGELPDSTL VQPTLDGPYQ PTSLNLPVDY WMLIAPTREG KVAEGTNTTD 100
Japan|Q01641| GHGELPDSTL VQPTLDGPYQ PTSLNLPVDY WMLIAPTREG KVAEGTNTTD 100
India|Q09113| GSGETLSGNV EEQPLDGPYT PDKSNLPSNY WYLINPSNDG VVFSVTDNST 100
Consensus GHGEINDSTT VEPVLDGPYQ PTTFKPPNDY WILINXNTNG VVYESTNNXD
100%
Conservation
0%
120
140
Japan|P13842| FWTAVVAVEP HVNPVDRQYT VFGENKQFNV RNDSD-KWKF LEMFRGSSQN 149
United States|B3SRX5| FWTAVVAVEP HVNPVDRQYT VFGENKQFNV RNDSD-KWKF LEMFRSSSQN 149
Japan|Q9WAK5| FWTVVVAVEP HVNPVDRQYT VFGENKQFNV RNDSD-KWKF LEMFRSSSQN 149
Japan|Q9WAK4| FWTAVVAVEP HVSPVDRQYT VFGENKQFNV RNDSD-KWKF LEMFRSSSQN 149
United States|P11193| FWTAVVAIEP HVNPVDRQYT IFGESKQFNV SNDSN-KWKF LEMFRSSSQN 149
United States|B3SRR9| FWTAVVAIEP HVNPVDRQYT IFGESKQFNV SNDSN-KWKF LEMFRSSSQN 149
Italy|P11194| FWTAVVAIEP HVNPVDRQYT IFGESKQFNV SNNTN-KWKF FEMFISSSQN 149
United States|P11195| FWTAVVAVEP HVNPVDRQYL IFGENKQFNV SNDSD-KWKF LEMFRSSSQN 149
United States|P11196| FWTAVSSVEP HVSQTNRQYI LFGENKQFNV ENNSD-KWKF FETFTGSSQG 149
Australia|P30214| FWTAVIAVEP HVSQTNRQYI LFGENKQFNV ENNSD-KWKF FEMFKGSSQG 149
Philippines|P21284| FWTAVIAVEP RVSQTNRQYI LFGENKQFNI ENNSD-KWKF FEMFKGSSQS 149
Bangladesh|ADW11134.1| FWTAVIAVEP HVSQTNRQYV LFGENKQFNI ENSSD-KWKF FEMFKGSSQS 149
Sweden|P11198| IWVALLLVEP NVTNQSRQYT LFGETKQITV ENNTN-KWKF FEMFRSSVSA 149
Australia|P11199| IWVALLLVEP NVTNQSRQYT LFGETKQITV ENNTN-KWKF FEMFRSSVSA 149
Venezuela|P11197| IWIALLLVEP NVTNQSRQYT LFGETKQITV ENNTN-KWKF FEMFRKNVSA 149
United Kingdom|P11200| IWIALLLVEP NVTNQSRQYT LFGETKQITI ENNTN-KWKF FEMFRSNVSS 149
Indonesia|P26451| RWLATILVEP GVASTTRTYT LFGIQEQITV ENSSNTKWKF IDLMKTTSSG 150
Israel|Q06895| RWLATILIEP NVTSQQRTYT IFGVQEQITI ENTSQTQWRF VDVSKTTQNG 150
Thailand|Q08778| RWFACVLVEL NVQNTQREYV LDGQTVQLQV SNDSSTLWKF ILFIKLEKNG 150
United kingdom|B3SRR1| RWFACVLVEP SVSNTQREYV LDGQTVQLQV SNDSSTLWKF ILFIKLGKNG 149
Belgium|Q3ZK58| RWFACVLVEP SVQSTRREYV LDGQTVQLQV SNDSSTLWKF ILFIKLEKNG 150
Japan|P39033| RWFACVLVEP NVQNTQRQYV LDGQNVQLQV SNDSSTSWKF ILFIKLTPDG 150
Japan|Q01641| RWFACVLVEP NVQNTQRQYV LDGQNVQLHV SNDSSTSWKF ILFIKLTPYG 150
India|Q09113| LWMFTYLVLP NTAQTSVVVN VMNETVNISI DN-SGSAYKF VDYFKTSSAQ 149
Consensus FWTAVVLVEP HVNXTDRQYT LFGENKQFNV ENDSD-KWKF FEMFXSSSQG
100%
Conservation
0%

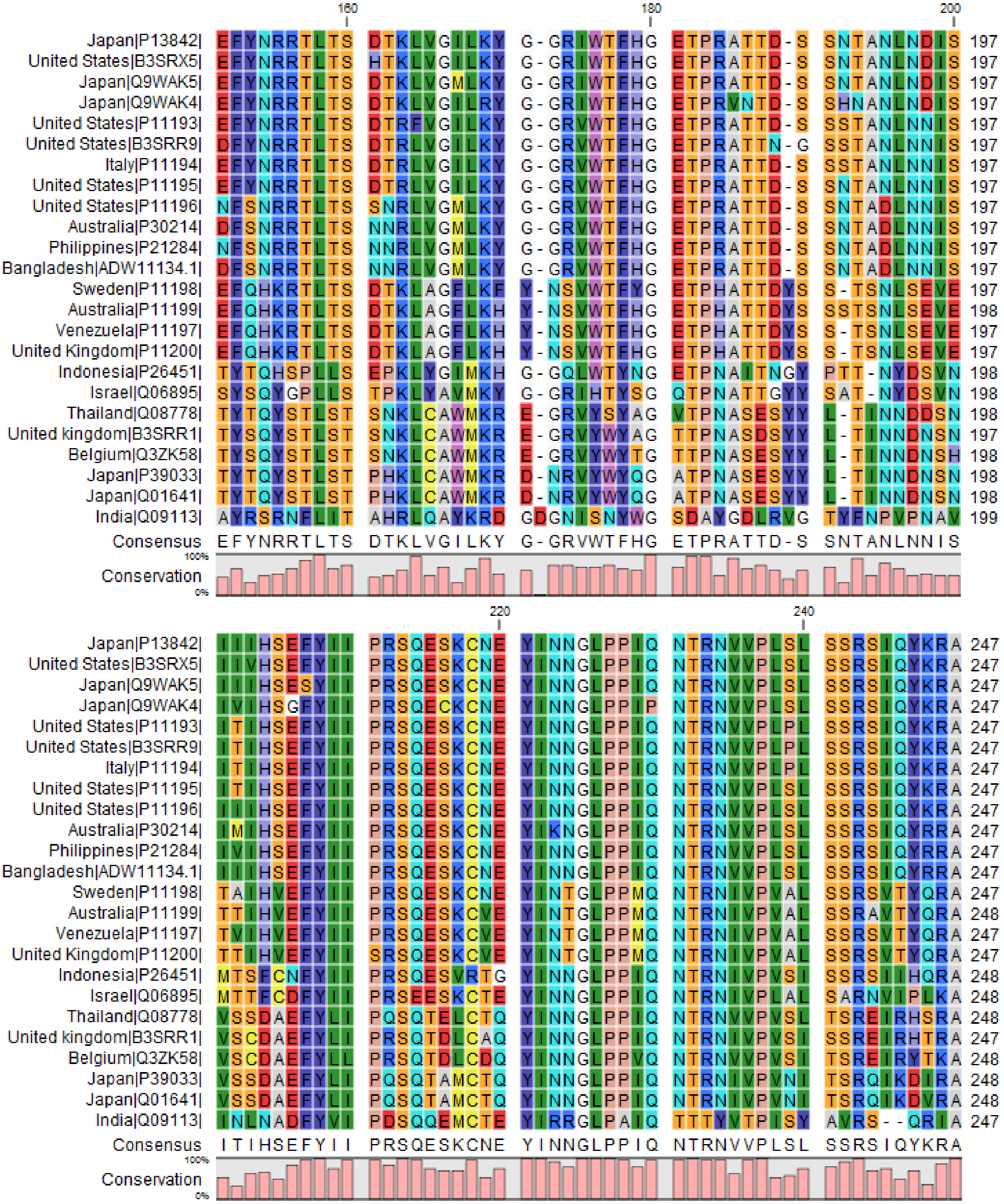

160
180
200
Japan|P13842| EFYNRRTLTS DTKLVGILKY G-GRIWTFHG ETPRATTD-S SNTANLNDIS 197
United States|B3SRX5| EFYNRRTLTS HTKLVGILKY G-GRIWTFHG ETPRATTD-S SNTANLNDIS 197
Japan|Q9WAK5| EFYNRRTLTS DTKLVGMLKY G-GRIWTFHG ETPRATTD-S SNTANLNDIS 197
Japan|Q9WAK4| EFYNRRTLTS DTKLVGILRY G-GRIWTFHG ETPRVNTD-S SHNANLNDIS 197
United States|P11193| EFYNRRTLTS DTRFVGILKY G-GRVWTFHG ETPRATTD-S SSTANLNNIS 197
United States|B3SRR9| DFYNRRTLTS DTRLVGILKY G-GRVWTFHG ETPRATTN-G SSTANLNNIS 197
Italy|P11194| EFYNRRTLTS DTRLVGILKY G-GRVWTFHG ETPRATTD-S SSTANLNNIS 197
United States|P11195| EFYNRRTLTS DTRLVGILKY G-GRVWTFHG ETPRATTD-S SNTANLNNIS 197
United States|P11196| NFSNRRTLTS SNRLVGMLKY G-GRVWTFHG ETPRATTD-S SNTADLNNIS 197
Australia|P30214| DFSNRRTLTS NNRLVGMLKY G-GRVWTFHG ETPRATTD-S SNTADLNNIS 197
Philippines|P21284| NFSNRRTLTS NNRLVGMLKY G-GRVWTFHG ETPRATTD-S SNTADLNNIS 197
Bangladesh|ADW11134.1| DFSNRRTLTS NNRLVGMLKY G-GRVWTFHG ETPRATTD-S SNTADLNNIS 197
Sweden|P11198| EFQHKRTLTS DTKLAGFLKF Y-NSVWTFYG ETPHATTDYS S-TSNLSEVE 197
Australia|P11199| EFQHKRTLTS DTKLAGFLKH Y-NSVWTFHG ETPHATTDYS SSTSNLSEVE 198
Venezuela|P11197| EFQHKRTLTS DTKLAGFLKH Y-NSVWTFHG ETPHATTDYS S-TSNLSEVE 197
United Kingdom|P11200| EFQHKRTLTS DTKLAGFLKH Y-NSVWTFHG ETPHATTDYS S-TSNLSEVE 197
Indonesia|P26451| TYTQHSPLLS EPKLYGIMKH G-GQLWTYNG ETPNAITNGY PTT-NYDSVN 198
Israel|Q06895| SYSQYGPLLS TPKLYAVMKY G-GRIHTYSG QTPNATTGYY SAT-NYDSVN 198
Thailand|Q08778| TYTQYSTLST SNKLCAWMKR E-GRVYSYAG VTPNASESYY L-TINNDDSN 198
United kingdom|B3SRR1| TYSQYSTLST SNKLCAWMKR E-GRVYWYAG TTPNASDSYY L-TINNDNSN 197
Belgium|Q3ZK58| TYSQYSTLST SNKLCAWMKR E-GRVYWYTG TTPNASESYY L-TINNDNSH 198
Japan|P39033| TYTQYSTLST PHKLCAWMKR D-NRVYWYQG ATPNASESYY L-TINNDNSN 198
Japan|Q01641| TYTQYSTLST PHKLCAWMKR D-NRVYWYQG ATPNASESYY L-TINNDNSN 198
India|Q09113| AYRSRNFLIT AHRLQAYKRD GDGNISNYWG SDAYGDLRVG TYFNPVPNAV 199
Consensus EFYNRRTLTS DTKLVGILKY G-GRVWTFHG ETPRATTD-S SNTANLNNIS
100%
Conservation
0%
220
240
Japan|P13842| IIIHSEFYII PRSQESKCNE YINNGLPPIQ NTRNVVPLSL SSRSIQYKRA 247
United States|B3SRX5| IIVHSEFYII PRSQESKCNE YINNGLPPIQ NTRNVVPLSL SSRSIQYKRA 247
Japan|Q9WAK5| IIIHSESYII PRSQESKCNE YINNGLPPIQ NTRNVVPLSL SSRSIQYKRA 247
Japan|Q9WAK4| IVIHSGFYII PRSQECKCNE YINNGLPPIP NTRNVVPLSL SSRSIQYKRA 247
United States|P11193| ITIHSEFYII PRSQESKCNE YINNGLPPIQ NTRNVVPLPL SSRSIQYKRA 247
United States|B3SRR9| ITIHSEFYII PRSQESKCNE YINNGLPPIQ NTRNVVPLPL SSRSIQYKRA 247
Italy|P11194| ITIHSEFYII PRSQESKCNE YINNGLPPIQ NTRNVVPLPL SSRSIQYKRA 247
United States|P11195| ITIHSEFYII PRSQESKCNE YINNGLPPIQ NTRNVVPLSL SSRSIQYKRA 247
United States|P11196| IIIHSEFYII PRSQESKCNE YINNGLPPIQ NTRNVVPLSL SSRSIQYKRA 247
Australia|P30214| IMIHSEFYII PRSQESKCNE YIKNGLPPIQ NTRNVVPLSL SSRSIQYRRA 247
Philippines|P21284| IVIHSEFYII PRSQESKCNE YINNGLPPIQ NTRNVVPLSL SSRSIQYRRA 247
Bangladesh|ADW11134.1| IIIHSEFYII PRSQESKCNE YINNGLPPIQ NTRNVVPLSL SSRSIQYRRA 247
Sweden|P11198| TAIHVEFYII PRSQESKCNE YINTGLPPMQ NTRNIVPVAL SSRSVTYQRA 247
Australia|P11199| TTIHVEFYII PRSQESKCVE YINTGLPPMQ NTRNIVPVAL SSRAVTYQRA 248
Venezuela|P11197| TVIHVEFYII PRSQESKCVE YINTGLPPMQ NTRNIVPVAL SSRSVTYQRA 247
United Kingdom|P11200| TTIHVEFYII SRSQESKCVE YINTGLPPMQ NTRNIVPVAL SSRSVTYQRA 247
Indonesia|P26451| MTSFCNFYII PRSQESVRTG YINNGLPPIQ NTRNIVPVSI SSRSIIHQRA 248
Israel|Q06895| MTTFCDFYII PRSEESKCTE YINNGLPPIQ NTRNIVPLAL SARNVIPLKA 248
Thailand|Q08778| VSSDAEFYLI PQSQTELCTQ YINNGLPPIQ NTRNVVPVSL TSREIRHSRA 248
United kingdom|B3SRR1| VSCDAEFYLI PRSQTDLCAQ YINNGLPPIQ NTRNIVPVSI SSREIRHTRA 247
Belgium|Q3ZK58| VSCDAEFYLL PRSQTDLCDQ YINNGLPPVQ NTRNVVPVSI TSREIRYTKA 248
Japan|P39033| VSSDAEFYLI PQSQTAMCTQ YINNGLPPIQ NTRNIVPVNI TSRQIKDIRA 248
Japan|Q01641| VSSDAEFYLI PQSQTAMCTQ YINNGLPPIQ NTRNIVPVNI TSRQIKDVRA 248
India|Q09113| INLNADFYVI PDSQQEMCTE YIRRGLPAIQ TTTYVTPISY AVRS--QRIA 247
Consensus ITIHSEFYII PRSQESKCNE YINNGLPPIQ NTRNVVPLSL SSRSIQYKRA
100%
Conservation
0%

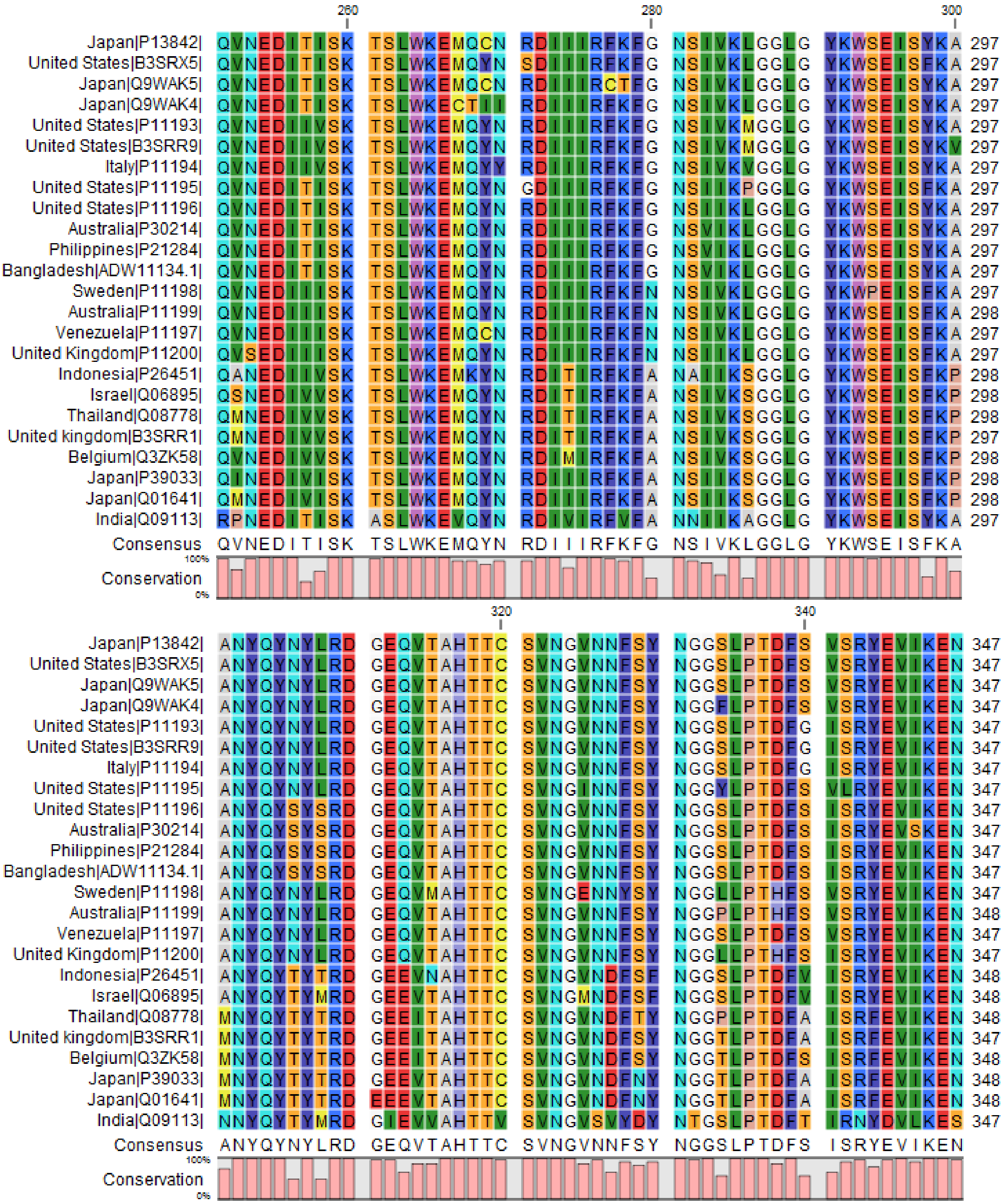
260 280 300
Japan|P13842| QVNEDITISK TSLWKEMQCN RDIIIRFKFG NSIVKLGGLG YKWSEISYKA 297
United States|B3SRX5| QVNEDITISK TSLWKEMQYN SDIIIRFKFG NSIVKLGGLG YKWSEISFKA 297
Japan|Q9WAK5| QVNEDITISK TSLWKEMQCN RDIIIRCTFG NSIVKLGGLG YKWSEISYKA 297
Japan|Q9WAK4| QVNEDITISK TSLWKECTII RDIIIRFKFG NSIVKLGGLG YKWSEISYKA 297
United States|P11193| QVNEDIIVSK TSLWKEMQYN RDIIIRFKFG NSIVKMGGLG YKWSEISYKA 297
United States|B3SRR9| QVNEDIIVSK TSLWKEMQYN RDIIIRFKFG NSIVKMGGLG YKWSEISYKV 297
Italy|P11194| QVNEDIIVSK TSLWKEMQYY RDIIIRFKFG NSIVKVGGLG YKWSEISYKA 297
United States|P11195| QVNEDITISK TSLWKEMQYN GDIIIRFKFG NSIIKPGGLG YKWSEISFKA 297
United States|P11196| QVNEDITISK TSLWKEMQYN RDIIIRFKFG NSIIKLGGLG YKWSEISYKA 297
Australia|P30214| QVNEDITISK TSLWKEMQYN RDIIIRFKFG NSVIKLGGLG YKWSEISYKA 297
Philippines|P21284| QVNEDITISK TSLWKEMQYN RDIIIRFKFG NSVIKLGGLG YKWSEISYKA 297
Bangladesh|ADW11134.1| QVNEDITISK TSLWKEMQYN RDIIIRFKFG NSVIKLGGLG YKWSEISYKA 297
Sweden|P11198| QVNEDIIISK TSLWKEMQYN RDIIIRFKFN NSIVKLGGLG YKWPEISFKA 297
Australia|P11199| QVNEDIIISK TSLWKEMQYN RDIIIRFKFN NSIVKLGGLG YKWSEISFKA 298
Venezuela|P11197| QVNEDIIISK TSLWKEMQCN RDIIIRFKFN NSIVKLGGLG YKWSEISFKA 297
United Kingdom|P11200| QVSEDIIISK TSLWKEMQYN RDIIIRFKFN NSIIKLGGLG YKWSEISFKA 297
Indonesia|P26451| QANEDIIVSK TSLWKEMKYN RDITIRFKFA NAIIKSGGLG YKWSEISFKP 298
Israel|Q06895| QSNEDIVVSK TSLWKEMQYN RDITIRFKFA NSIVKSGGLG YKWSEISFKP 298
Thailand|Q08778| QMNEDIVVSK TSLWKEMQYN RDITIRFKFA NSIVKSGGLG YKWSEISFKP 298
United kingdom|B3SRR1| QMNEDIVVSK TSLWKEMQYN RDITIRFKFA NSIVKSGGLG YKWSEISFKP 297
Belgium|Q3ZK58| QVNEDIVVSK TSLWKEMQYN RDIMIRFKFA NSIVKSGGLG YKWSEISFKP 298
Japan|P39033| QINEDIVISK TSLWKEMQYN RDIIIRFKFA NSIIKSGGLG YKWSEISFKP 298
Japan|Q01641| QMNEDIVISK TSLWKEMQYN RDIIIRFKFA NSIIKSGGLG YKWSEISFKP 298
India|Q09113| RPNEDITISK ASLWKEVQYN RDIVIRFVFA NNIIKAGGLG YKWSEISYKA 297
Consensus QVNEDITISK TSLWKEMQYN RDIIIRFKFG NSIVKLGGLG YKWSEISFKA
100%
Conservation
0%
320 340
Japan|P13842| ANYQYNYLRD GEQVTAHTTC SVNGVNNFSY NGGSLPTDFS VSRYEVIKEN 347
United States|B3SRX5| ANYQYNYLRD GEQVTAHTTC SVNGVNNFSY NGGSLPTDFS VSRYEVIKEN 347
Japan|Q9WAK5| ANYQYNYLRD GEQVTAHTTC SVNGVNNFSY NGGSLPTDFS VSRYEVIKEN 347
Japan|Q9WAK4| ANYQYNYLRD GEQVTAHTTC SVNGVNNFSY NGGFLPTDFS VSRYEVIKEN 347
United States|P11193| ANYQYNYLRD GEQVTAHTTC SVNGVNNFSY NGGSLPTDFG ISRYEVIKEN 347
United States|B3SRR9| ANYQYNYLRD GEQVTAHTTC SVNGVNNFSY NGGSLPTDFG ISRYEVIKEN 347
Italy|P11194| ANYQYNYLRD GEQVTAHTTC SVNGVNNFSY NGGSLPTDFG ISRYEVIKEN 347
United States|P11195| ANYQYNYLRD GEQVTAHTTC SVNGINNFSY NGGYLPTDFS VLRYEVIKEN 347
United States|P11196| ANYQYSYSRD GEQVTAHTTC SVNGVNNFSY NGGSLPTDFS ISRYEVIKEN 347
Australia|P30214| ANYQYSYSRD GEQVTAHTTC SVNGVNNFSY NGGSLPTDFS ISRYEVSKEN 347
Philippines|P21284| ANYQYSYSRD GEQVTAHTTC SVNGVNNFSY NGGSLPTDFS ISRYEVIKEN 347
Bangladesh|ADW11134.1| ANYQYSYSRD GEQVTAHTTC SVNGVNNFSY NGGSLPTDFS ISRYEVIKEN 347
Sweden|P11198| ANYQYNYLRD GEQVMAHTTC SVNGENNYSY NGGLLPTHFS VSRYEVIKEN 347
Australia|P11199| ANYQYNYLRD GEQVTAHTTC SVNGVNNFSY NGGPLPTHFS VSRYEVIKEN 348
Venezuela|P11197| ANYQYNYLRD GEQVTAHTTC SVNGVNNFSY NGGSLPTDFS VSRYEVIKEN 347
United Kingdom|P11200| ANYQYNYLRD GEQVTAHTTC SVNGVNNFSY NGGLLPTHFS ISRYEVIKEN 347
Indonesia|P26451| ANYQYTYTRD GEEVNAHTTC SVNGVNDFSF NGGSLPTDFV ISRYEVIKEN 348
Israel|Q06895| ANYQYTYMRD GEEVTAHTTC SVNGMNDFSF NGGSLPTDFV ISRYEVIKEN 348
Thailand|Q08778| MNYQYTYTRD GEEITAHTTC SVNGVNDFTY NGGPLPTDFA ISRFEVIKEN 348
United kingdom|B3SRR1| MNYQYTYTRD GEEITAHTTC SVNGVNDFSY NGGTLPTDFA ISRFEVIKEN 347
Belgium|Q3ZK58| MNYQYTYTRD GEEITAHTTC SVNGVNDFSY NGGTLPTDFS ISRFEVIKEN 348
Japan|P39033| MNYQYTYTRD GEEVTAHTTC SVNGVNDFNY NGGTLPTDFA ISRFEVIKEN 348
Japan|Q01641| MNYQYTYTRD EEEVTAHTTC SVNGVNDFNY NGGTLPTDFA ISRFEVIKEN 348
India|Q09113| NNYQYTYMRD GIEVVAHTTV SVNGVSVYDY NTGSLPTDFT IRNYDVLKES 347
Consensus ANYQYNYLRD GEQVTAHTTC SVNGVNNFSY NGGSLPTDFS ISRYEVIKEN
100%
Conservation
0%

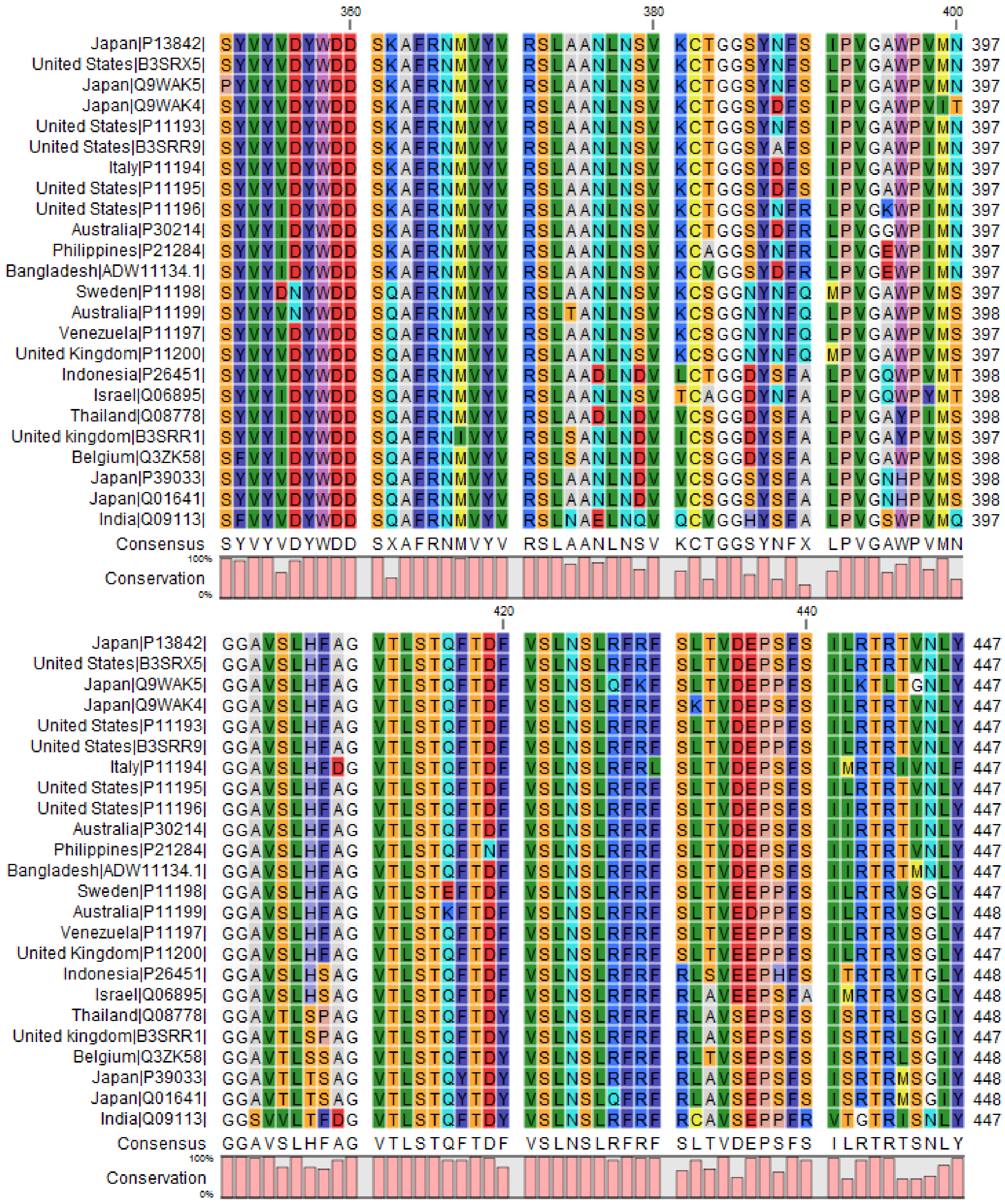
360
380
400
Japan|P13842| SYVYVDYWDD SKAFRNMVYV RSLAANLNSV KCTGGSYNFS IPVGAWPVMN 397
United States|B3SRX5| SYVYVDYWDD SKAFRNMVYV RSLAANLNSV KCTGGSYNFS LPVGAWPVMN 397
Japan|Q9WAK5| PYVYVDYWDD SKAFRNMVYV RSLAANLNSV KCTGGSYNFS LPVGAWPVMN 397
Japan|Q9WAK4| SYVYVDYWDD SKAFRNMVYV RSLAANLNSV KCTGGSYDFS IPVGAWPVIT 397
United States|P11193| SYVYVDYWDD SKAFRNMVYV RSLAANLNSV KCTGGSYNFS IPVGAWPVMN 397
United States|B3SRR9| SYVYVDYWDD SKAFRNMVYV RSLAANLNSV KCTGGSYAFS IPVGAWPIMN 397
Italy|P11194| SYVYVDYWDD SKAFRNMVYV RSLAANLNSV KCTGGSYDFS IPVGAWPVMN 397
United States|P11195| SYVYVDYWDD SKAFRNMVYV RSLAANLNSV KCTGGSYDFS IPVGAWPVMN 397
United States|P11196| SYVYIDYWDD SKAFRNMVYV RSLAANLNSV KCTGGSYNFR LPVGKWPIMN 397
Australia|P30214| SYVYIDYWDD SKAFRNMVYV RSLAANLNSV KCTGGSYDFR LPVGGWPIMN 397
Philippines|P21284| SYVYIDYWDD SKAFRNMVYV RSLAANLNSV KCAGGSYNFR LPVGEWPIMN 397
Bangladesh|ADW11134.1| SYVYIDYWDD SKAFRNMVYV RSLAANLNSV KCVGGSYDFR LPVGEWPIMN 397
Sweden|P11198| SYVYDNYWDD SQAFRNMVYV RSLAANLNSV KCSGGNYNFQ MPVGAWPVMS 397
Australia|P11199| SYVYVNYWDD SQAFRNMVYV RSLTANLNSV KCSGGNYNFQ LPVGAWPVMS 398
Venezuela|P11197| SYVYVDYWDD SQAFRNMVYV RSLAANLNSV KCSGGNYNFQ LPVGAWPVMS 397
United Kingdom|P11200| SYVYVDYWDD SQAFRNMVYV RSLAANLNSV KCSGGNYNFQ MPVGAWPVMS 397
Indonesia|P26451| SYVYVDYWDD SQAFRNMVYV RSLAADLNDV LCTGGDYSFA LPVGQWPVMT 398
Israel|Q06895| SYVYIDYWDD SQAFRNMVYV RSLAANLNSV TCAGGDYNFA LPVGQWPYMT 398
Thailand|Q08778| SYVYIDYWDD SQAFRNMVYV RSLAADLNDV VCSGGDYSFA LPVGAYPIMS 398
United kingdom|B3SRR1| SYVYIDYWDD SQAFRNIVYV RSLSANLNDV ICSGGDYSFA LPVGAYPVMS 397
Belgium|Q3ZK58| SFVYIDYWDD SQAFRNMVYV RSLSANLNDV VCSGGDYSFA LPVGAWPVMS 398
Japan|P39033| SYVYVDYWDD SQAFRNMVYV RSLAANLNDV VCSGGSYSFA LPVGNHPVMS 398
Japan|Q01641| SYVYVDYWDD SQAFRNMVYV RSLAANLNDV VCSGGSYSFA LPVGNHPVMS 398
India|Q09113| SFVYVDYWDD SQAFRNMVYV RSLNAELNQV QCVGGHYSFA LPVGSWPVMQ 397
Consensus SYVYVDYWDD SXAFRNMVYV RSLAANLNSV KCTGGSYNFX LPVGAWPVMN
100%
Conservation
0%
420
440
Japan|P13842| GGAVSLHFAG VTLSTQFTDF VSLNSLRFRF SLTVDEPSFS ILRTRTVNLY 447
United States|B3SRX5| GGAVSLHFAG VTLSTQFTDF VSLNSLRFRF SLTVDEPSFS ILRTRTVNLY 447
Japan|Q9WAK5| GGAVSLHFAG VTLSTQFTDF VSLNSLQFKF SLTVDEPPFS ILKTLTGNLY 447
Japan|Q9WAK4| GGAVSLHFAG VTLSTQFTDF VSLNSLRFRF SKTVDEPSFS ILRTRTVNLY 447
United States|P11193| GGAVSLHFAG VTLSTQFTDF VSLNSLRFRF SLTVDEPPFS ILRTRTVNLY 447
United States|B3SRR9| GGAVSLHFAG VTLSTQFTDF VSLNSLRFRF SLTVDEPPFS ILRTRTVNLY 447
Italy|P11194| GGAVSLHFDG VTLSTQFTDF VSLNSLRFRL SLTVDEPSFS IMRTRIVNLF 447
United States|P11195| GGAVSLHFAG VTLSTQFTDF VSLNSLRFRF SLTVDEPSFS ILRTRTVNLY 447
United States|P11196| GGAVSLHFAG VTLSTQFTDF VSLNSLRFRF SLTVDEPSFS IIRTRTINLY 447
Australia|P30214| GGAVSLHFAG VTLSTQFTDF VSLNSLRFRF SLTVDEPSFS IIRTRTINLY 447
Philippines|P21284| GGAVSLHFAG VTLSTQFTNF VSLNSLRFRF SLTVDEPSFS IIRTRTVNLY 447
Bangladesh|ADW11134.1| GGAVSLHFAG VTLSTQFTDF VSLNSLRFRF SLTVDEPSFS IIRTRTMNLY 447
Sweden|P11198| GGAVSLHFAG VTLSTEFTDF VSLNSLRFRF SLTVEEPPFS ILRTRVSGLY 447
Australia|P11199| GGAVSLHFAG VTLSTKFTDF VSLNSLRFRF SLTVEDPPFS ILRTRVSGLY 448
Venezuela|P11197| GGAVSLHFAG VTLSTQFTDF VSLNSLRFRF SLTVEEPPFS ILRTRVSGLY 447
United Kingdom|P11200| GGAVSLHFAG VTLSTQFTDF VSLNSLRFRF SLTVEEPPFS ILRTRVSGLY 447
Indonesia|P26451| GGAVSLHSAG VTLSTQFTDF VSLNSLRFRF RLSVEEPHFS ITRTRVTGLY 448
Israel|Q06895| GGAVSLHSAG VTLSTQFTDF VSLNSLRFRF RLAVEEPSFA IMRTRVSGLY 448
Thailand|Q08778| GGAVTLSPAG VTLSTQFTDY VSLNSLRFRF RLAVSEPSFS ISRTRLSGIY 448
United kingdom|B3SRR1| GGAVTLSPAG VTLSTQFTDY VSLNSLRFRF RLAVSEPSFS ISRTRLSGIY 447
Belgium|Q3ZK58| GGAVTLSSAG VTLSTQFTDY VSLNSLRFRF RLTVSEPSFS ISRTRLSGIY 448
Japan|P39033| GGAVTLTSAG VTLSTQYTDY VSLNSLRFRF RLAVSEPSFS ISRTRMSGIY 448
Japan|Q01641| GGAVTLTSAG VTLSTQYTDY VSLNSLQFRF RLAVSEPSFS ISRTRMSGIY 448
India|Q09113| GGSVVLTFDG VTLSTQFTDY VSLNSLRFRF RCAVSEPPFR VTGTRISNLY 447
Consensus GGAVSLHFAG VTLSTQFTDF VSLNSLRFRF SLTVDEPSFS ILRTRTSNLY
100%
Conservation
0%

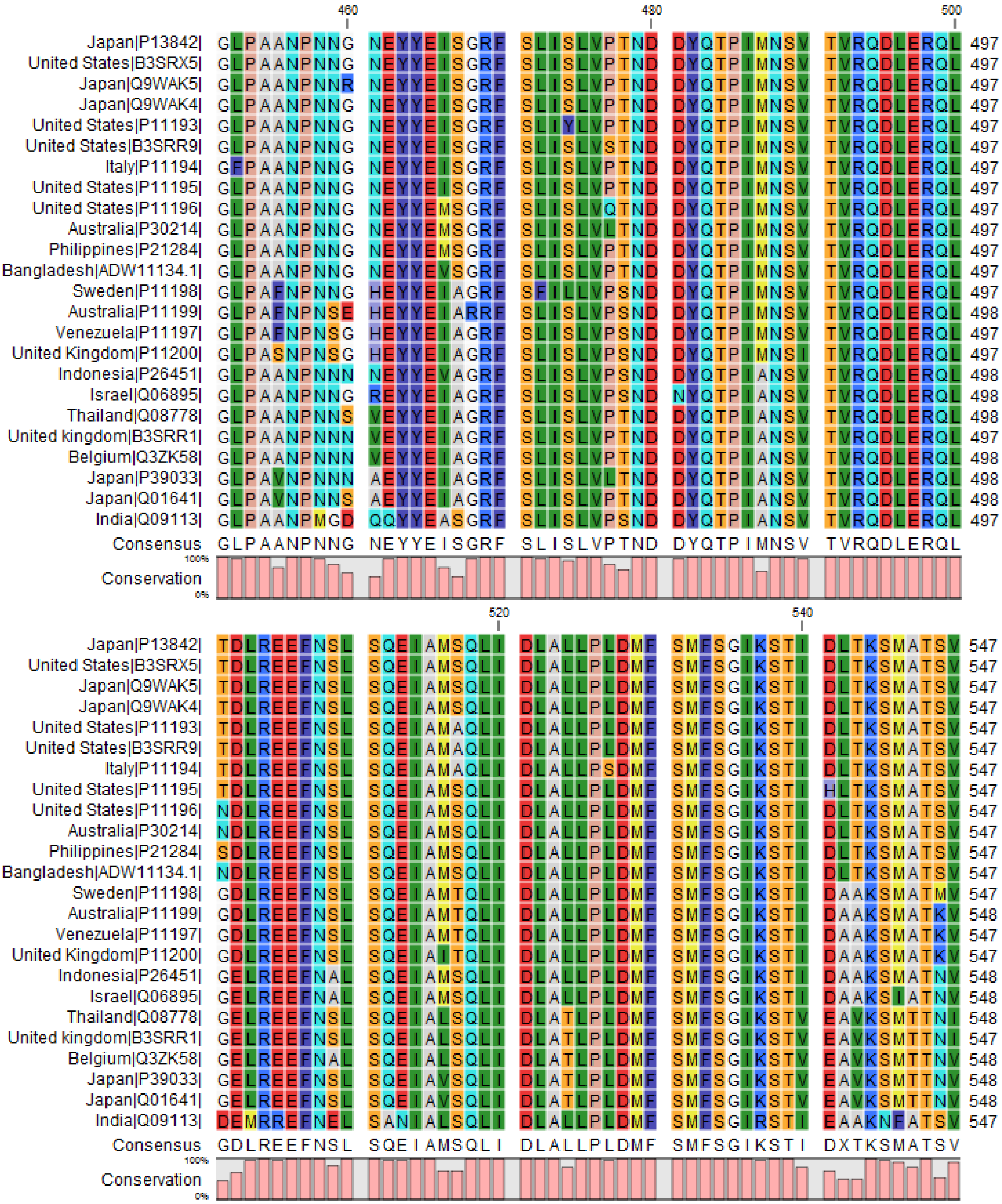
460 480 500
Japan|P13842| GLPAANPNNG NEYYEISGRF SLISLVPTND DYQTPIMNSV TVRQDLERQL 497
United States|B3SRX5| GLPAANPNNG NEYYEISGRF SLISLVPTND DYQTPIMNSV TVRQDLERQL 497
Japan|Q9WAK5| GLPAANPNNR NEYYEISGRF SLISLVPTND DYQTPIMNSV TVRQDLERQL 497
Japan|Q9WAK4| GLPAANPNNG NEYYEISGRF SLISLVPTND DYQTPIMNSV TVRQDLERQL 497
United States|P11193| GLPAANPNNG NEYYEISGRF SLIYLVPTND DYQTPIMNSV TVRQDLERQL 497
United States|B3SRR9| GLPAANPNNG NEYYEISGRF SLISLVSTND DYQTPIMNSV TVRQDLERQL 497
Italy|P11194| GFPAANPNNG NEYYEISGRF SLISLVPTND DYQTPIMNSV TVRQDLERQL 497
United States|P11195| GLPAANPNNG NEYYEISGRF SLISLVPTND DYQTPIMNSV TVRQDLERQL 497
United States|P11196| GLPAANPNNG NEYYEMSGRF SLISLVQTND DYQTPIMNSV TVRQDLERQL 497
Australia|P30214| GLPAANPNNG NEYYEMSGRF SLISLVLTND DYQTPIMNSV TVRQDLERQL 497
Philippines|P21284| GLPAANPNNG NEYYEMSGRF SLISLVPTND DYQTPIMNSV TVRQDLERQL 497
Bangladesh|ADW11134.1| GLPAANPNNG NEYYEVSGRF SLISLVPTND DYQTPIMNSV TVRQDLERQL 497
Sweden|P11198| GLPAFNPNNG HEYYEIAGRF SFILLVPSND DYQTPIMNSV TVRQDLERQL 497
Australia|P11199| GLPAFNPNSE HEYYEIARRF SLISLVPSND DYQTPIMNSV TVRQDLERQL 498
Venezuela|P11197| GLPAFNPNSG HEYYEIAGRF SLISLVPSND DYQTPIMNSV TVRQDLERQL 497
United Kingdom|P11200| GLPASNPNSG HEYYEIAGRF SLISLVPSND DYQTPIMNSI TVRQDLERQL 497
Indonesia|P26451| GLPAANPNNN NEYYEVAGRF SLISLVPSND DYQTPIANSV TVRQDLERQL 498
Israel|Q06895| GLPAANPNNG REYYEIAGRF SLISLVPSND NYQTPIANSV TVRQDLERQL 498
Thailand|Q08778| GLPAANPNNS VEYYEIAGRF SLISLVPTND DYQTPIANSV TVRQDLERQL 498
United kingdom|B3SRR1| GLPAANPNNN VEYYEIAGRF SLISLVPTND DYQTPIANSV TVRQDLERQL 497
Belgium|Q3ZK58| GLPAANPNNN VEYYEIAGRF SLISLVPTND DYQTPIANSV TVRQDLERQL 498
Japan|P39033| GLPAVNPNNN AEYYEIAGRF SLISLVLTND DYQTPIANSV TVRQDLERQL 498
Japan|Q01641| GLPAVNPNNS AEYYEIAGRF SLISLVPTND DYQTPIANSV TVRQDLERQL 498
India|Q09113| GLPAANPMGD QQYYEASGRF SLISLVPSND DYQTPIANSV TVRQDLERQL 497
Consensus GLPAANPNNG NEYYEISGRF SLISLVPTND DYQTPIMNSV TVRQDLERQL
100%
Conservation
0%
520 540
Japan|P13842| TDLREEFNSL SQEIAMSQLI DLALLPLDMF SMFSGIKSTI DLTKSMATSV 547
United States|B3SRX5| TDLREEFNSL SQEIAMSQLI DLALLPLDMF SMFSGIKSTI DLTKSMATSV 547
Japan|Q9WAK5| TDLREEFNSL SQEIAMSQLI DLALLPLDMF SMFSGIKSTI DLTKSMATSV 547
Japan|Q9WAK4| TDLREEFNSL SQEIAMSQLI DLALLPLDMF SMFSGIKSTI DLTKSMATSV 547
United States|P11193| TDLREEFNSL SQEIAMAQLI DLALLPLDMF SMFSGIKSTI DLTKSMATSV 547
United States|B3SRR9| TDLREEFNSL SQEIAMAQLI DLALLPLDMF SMFSGIKSTI DLTKSMATSV 547
Italy|P11194| TDLREEFNSL SQEIAMAQLI DLALLPSDMF SMFSGIKSTI DLTKSMATSV 547
United States|P11195| TDLREEFNSL SQEIAMSQLI DLALLPLDMF SMFSGIKSTI HLTKSMATSV 547
United States|P11196| NDLREEFNSL SQEIAMSQLI DLALLPLDMF SMFSGIKSTI DLTKSMATSV 547
Australia|P30214| NDLREEFNSL SQEIAMSQLI DLALLPLDMF SMFSGIKSTI DLTKSMATSV 547
Philippines|P21284| SDLREEFNSL SQEIAMSQLI DLALLPLDMF SMFSGIKSTI DLTKSMATSV 547
Bangladesh|ADW11134.1| NDLREEFNSL SQEIAMSQLI DLALLPLDMF SMFSGIKSTI DLTKSMATSV 547
Sweden|P11198| GDLREEFNSL SQEIAMTQLI DLALLPLDMF SMFSGIKSTI DAAKSMATMV 547
Australia|P11199| GDLREEFNSL SQEIAMTQLI DLALLPLDMF SMFSGIKSTI DAAKSMATKV 548
Venezuela|P11197| GDLREEFNSL SQEIAMTQLI DLALLPLDMF SMFSGIKSTI DAAKSMATKV 547
United Kingdom|P11200| GDLREEFNSL SQEIAITQLI DLALLPLDMF SMFSGIKSTI DAAKSMATKV 547
Indonesia|P26451| GELREEFNAL SQEIAMSQLI DLALLPLDMF SMFSGIKSTI DAAKSMATNV 548
Israel|Q06895| GELREEFNAL SQEIAMSQLI DLALLPLDMF SMFSGIKSTI DAAKSIATNV 548
Thailand|Q08778| GELREEFNSL SQEIALSQLI DLATLPLDMF SMFSGIKSTV EAVKSMTTNI 548
United kingdom|B3SRR1| GELREEFNSL SQEIALSQLI DLATLPLDMF SMFSGIKSTV EAVKSMTTNI 547
Belgium|Q3ZK58| GELREEFNAL SQEIALSQLI DLATLPLDMF SMFSGIKSTV EAVKSMTTNV 548
Japan|P39033| GELREEFNSL SQEIAVSQLI DLATLPLDMF SMFSGIKSTV EAVKSMTTNV 548
Japan|Q01641| GELREEFNSL SQEIAVSQLI DLATLPLDMF SMFSGIKSTV EAVKSMTTNV 548
India|Q09113| DEMRREFNEL SANIALSQLI DLALLPLDMF SMFSGIRSTI EAAKNFATSV 547
Consensus GDLREEFNSL SQEIAMSQLI DLALLPLDMF SMFSGIKSTI DXTKSMATSV
100%
Conservation
0%

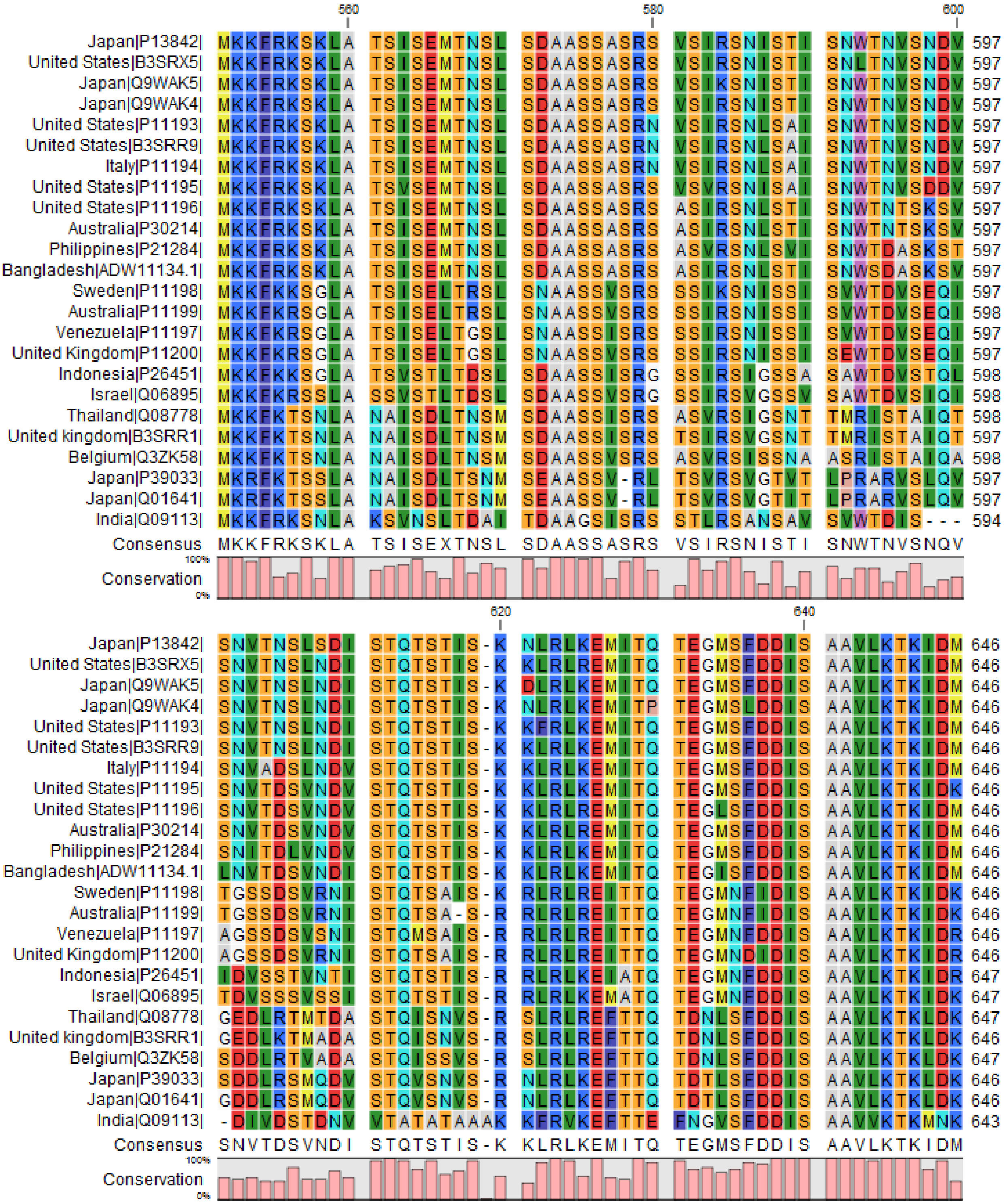

560
580
600
Japan|P13842|
United States|B3SRX5|
Japan|Q9WAK5|
Japan|Q9WAK4|
United States|P11193|
United States|B3SRR9|
Italy|P11194|
United States|P11195|
United States|P11196|
Australia|P30214|
Philippines|P21284|
Bangladesh|ADW11134.1|
Sweden|P11198|
Australia|P11199|
Venezuela|P11197|
United Kingdom|P11200|
Indonesia|P26451|
Israel|Q06895|
Thailand|Q08778|
United kingdom|B3SRR1|
Belgium|Q3ZK58|
Japan|P39033|
Japan|Q01641|
India|Q09113|
Consensus MKKFRKSKLA TSISEXTNSL SDAASSASRS VSIRSNISTI SNWTNVSNQV
Conservation
100%
0%
620
640
Consensus SNVTDSVNDI STQTSTIS-K KLRLKEMITQ TEGMSFDDIS AAVLKTKIDM
Conservation

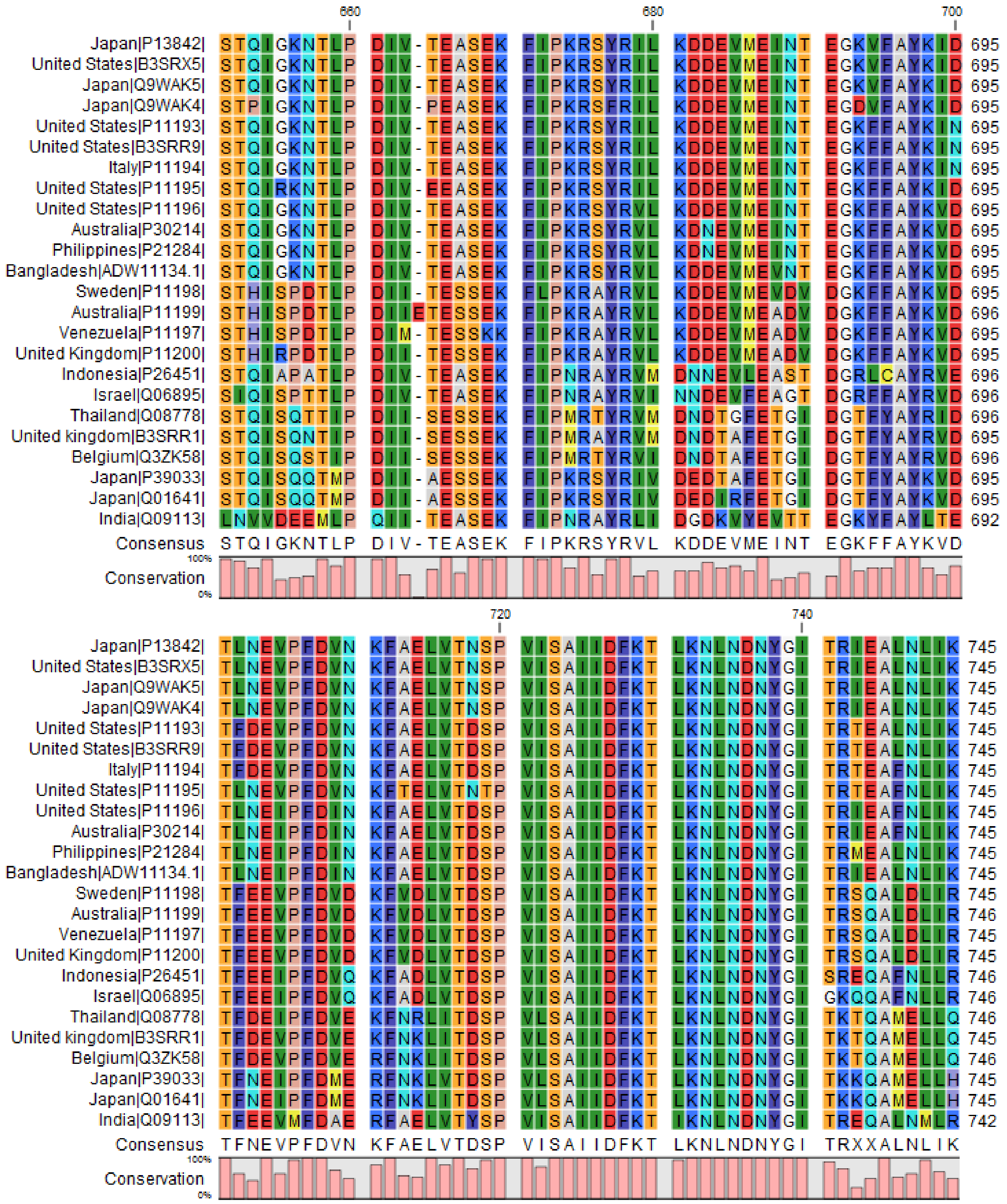
660
680
700
Japan|P13842| STQIGKNTLP DIV-TEASEK FIPKRSYRIL KDDEVMEINT EGKVFAYKID 695
United States|B3SRX5| STQIGKNTLP DIV-TEASEK FIPKRSYRIL KDDEVMEINT EGKVFAYKID 695
Japan|Q9WAK5| STQIGKNTLP DIV-TEASEK FIPKRSYRIL KDDEVMEINT EGKVFAYKID 695
Japan|Q9WAK4| STPIGKNTLP DIV-PEASEK FIPKRSFRIL KDDEVMEINT EGDVFAYKID 695
United States|P11193| STQIGKNTLP DIV-TEASEK FIPKRSYRIL KDDEVMEINT EGKFFAYKIN 695
United States|B3SRR9| STQIGKNTLP DIV-TEASEK FIPKRSYRIL KDDEVMEINT EGKFFAYKIN 695
Italy|P11194| STQIGKNTLP DIV-TEASEK FIPKRSYRIL KDDEVMEINT EGKFFAYKIN 695
United States|P11195| STQIRKNTLP DIV-EEASEK FIPKRSYRIL KDDEVMEINT EGKFFAYKID 695
United States|P11196| STQIGKNTLP DIV-TEASEK FIPKRSYRVL KDDEVMEINT EGKFFAYKVD 695
Australia|P30214| STQIGKNTLP DIV-TEASEK FIPKRSYRVL KDNEVMEINT EGKFFAYKVD 695
Philippines|P21284| STQIGKNTLP DIV-TEASEK FIPKRSYRVL KDNEVMEINT EGKFFAYKVD 695
Bangladesh|ADW11134.1| STQIGKNTLP DIV-TEASEK FIPKRSYRVL KDDEVMEVNT EGKFFAYKVD 695
Sweden|P11198| STHISPDTLP DII-TESSEK FLPKRAYRVL KDDEVMEVDV DGKFFAYKVD 695
Australia|P11199| STHISPDTLP DIIETESSEK FIPKRAYRVL KDDEVMEADV DGKFFAYKVD 696
Venezuela|P11197| STHISPDTLP DIM-TESSKK FIPKRAYRVL KDDEVMEADV DGKFFAYKVD 695
United Kingdom|P11200| STHIRPDTLP DII-TESSEK FIPKRAYRVL KDDEVMEADV DGKFFAYKVD 695
Indonesia|P26451| STQIAPATLP DIV-TEASEK FIPNRAYRVM DNNEVLEAST DGRLCAYRVE 696
Israel|Q06895| SIQISPTTLP DIV-TEASEK FIPNRAYRVI NNDEVFEAGT DGRFFAYRVD 696
Thailand|Q08778| STQISQTTIP DII-SESSEK FIPMRTYRVM DNDTGFETGI DGTFYAYRID 696
United kingdom|B3SRR1| STQISQNTIP DII-SESSEK FIPMRAYRVM DNDTAFETGI DGTFYAYRVD 695
Belgium|Q3ZK58| STQISQSTIP DII-SESSEK FIPMRTYRVI DNDTAFETGI DGTFYAYRVD 696
Japan|P39033| STQISQQTMP DII-AESSEK FIPKRSYRIV DEDTAFETGI DGTFYAYKVD 695
Japan|Q01641| STQISQQTMP DII-AESSEK FIPKRSYRIV DEDIRFETGI DGTFYAYKVD 695
India|Q09113| LNVVDEEMLP QII-TEASEK FIPNRAYRLI DGDKVYEVTT EGKYFAYLTE 692
Consensus STQIGKNTLP DIV-TEASEK FIPKRSYRVL KDDEVMEINT EGKFFAYKVD
100%
Conservation
0%
720
740
Japan|P13842| TLNEVPFDVN KFAELVTNSP VISAIIDFKT LKNLNDNYGI TRIEALNLIK 745
United States|B3SRX5| TLNEVPFDVN KFAELVTNSP VISAIIDFKT LKNLNDNYGI TRIEALNLIK 745
Japan|Q9WAK5| TLNEVPFDVN KFAELVTNSP VISAIIDFKT LKNLNDNYGI TRIEALNLIK 745
Japan|Q9WAK4| TLNEVPFDVN KFAELVTNSP VISAIIDFKT LKNLNDNYGI TRIEALNLIK 745
United States|P11193| TFDEVPFDVN KFAELVTDSP VISAIIDFKT LKNLNDNYGI TRTEALNLIK 745
United States|B3SRR9| TFDEVPFDVN KFAELVTDSP VISAIIDFKT LKNLNDNYGI TRTEALNLIK 745
Italy|P11194| TFDEVPFDVN KFAELVTDSP VISAIIDFKT LKNLNDNYGI TRTEAFNLIK 745
United States|P11195| TLNEVPFDVN KFTELVTNTP VISAIIDFKT LKNLNDNYGI TRTEAFNLIK 745
United States|P11196| TLNEIPFDIN KFAELVTDSP VISAIIDFKT LKNLNDNYGI TRIEAFNLIK 745
Australia|P30214| TLNEIPFDIN KFAELVTDSP VISAIIDFKT LKNLNDNYGI TRIEAFNLIK 745
Philippines|P21284| TLNEIPFDIN KFAELVTDSP VISAIIDFKT LKNLNDNYGI TRMEALNLIK 745
Bangladesh|ADW11134.1| TLNEIPFDIN KFAELVTDSP VISAIIDFKT LKNLNDNYGI TRIEALNLIK 745
Sweden|P11198| TFEEVPFDVD KFVDLVTDSP VISAIIDFKT LKNLNDNYGI TRSQALDLIR 745
Australia|P11199| TFEEVPFDVD KFVDLVTDSP VISAIIDFKT LKNLNDNYGI TRSQALDLIR 746
Venezuela|P11197| TFEEVPFDVD KFVDLVTDSP VISAIIDFKT LKNLNDNYGI TRSQALDLIR 745
United Kingdom|P11200| TFEEVPFDVD KFVDLVTDSP VISAIIDFKT LKNLNDNYGI TRSQALDLIR 745
Indonesia|P26451| TFEEIPFDVQ KFADLVTDSP VISAIIDFKT LKNLNDNYGI SREQAFNLLR 746
Israel|Q06895| TFEEIPFDVQ KFADLVTDSP VISAIIDFKT LKNLNDNYGI GKQQAFNLLR 746
Thailand|Q08778| TFDEIPFDVE KFNRLITDSP VLSAIIDFKT LKNLNDNYGI TKTQAMELLQ 746
United kingdom|B3SRR1| TFDEVPFDVE KFNKLITDSP VLSAIIDFKT LKNLNDNYGI TKTQAMELLQ 745
Belgium|Q3ZK58| TFDEVPFDVE RFNKLITDSP VLSAIIDFKT LKNLNDNYGI TKTQAMELLQ 746
Japan|P39033| TFNEIPFDME RFNKLVTDSP VLSAIIDFKT LKNLNDNYGI TKKQAMELLH 745
Japan|Q01641| TFNEIPFDME RFNKLITDSP VLSAIIDFKT LKNLNDNYGI TKKQAMELLH 745
India|Q09113| TFEEVMFDAE RFAELVTYSP VISAIIDFKT IKNLNDNYGI TREQALNMLR 742
Consensus TFNEVPFDVN KFAELVTDSP VISAIIDFKT LKNLNDNYGI TRXXALNLIK
100%
Conservation
0%

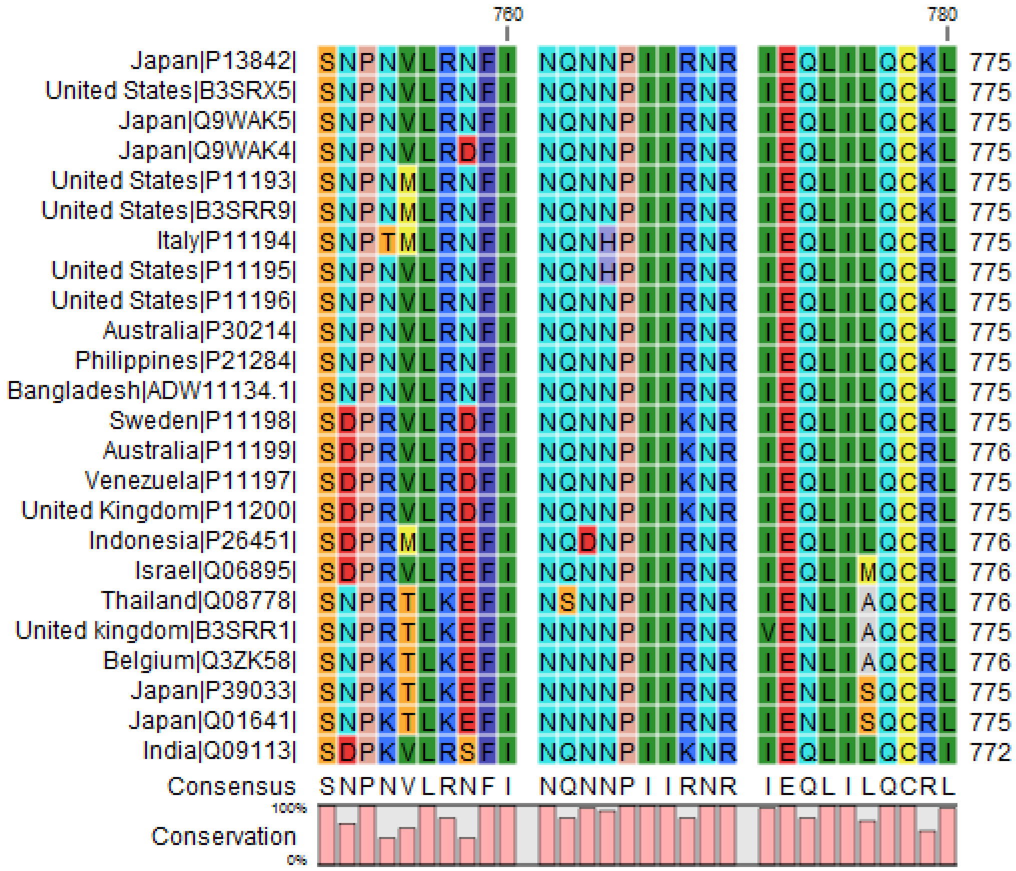

S1 Fig: Alignment of available twenty four VP4 protein sequences of human rotavirus A retrieved from databases.
