# Supplementary figures and images for: Therapeutics Insight with Inclusive Immunopharmacology Explication of Human Rotavirus A for the Treatment of Diarrhea
Source: Front Pharmacol. 2016 Jun 23;7:153. doi: 10.3389/fphar.2016.00153 (PMC4917548; doi:10.3389/fphar.2016.00153)

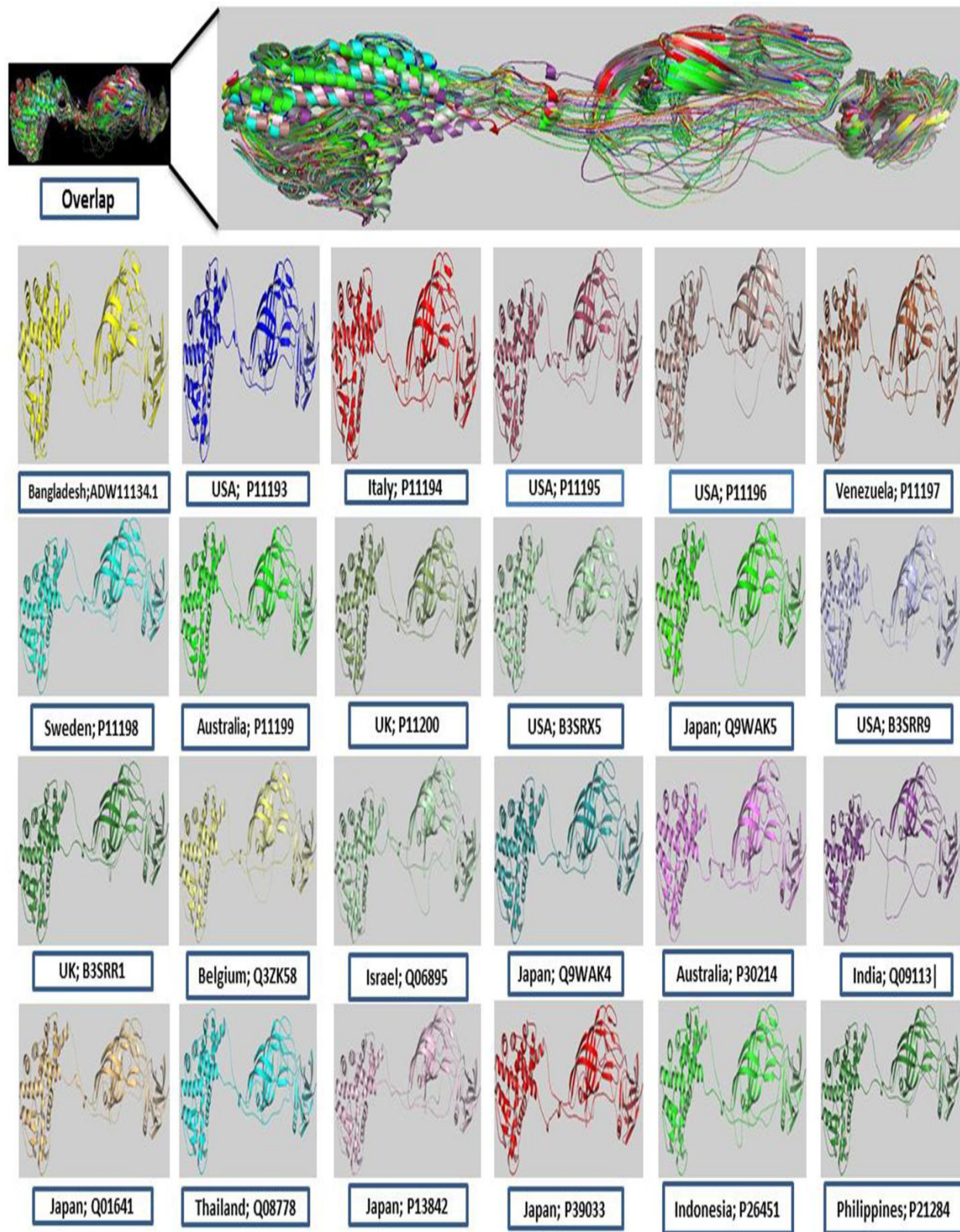

**S2 Fig: Predicted 3D model of all VP4 strains.**

Supplement: Supplementary file 3 [file Image2.PDF]

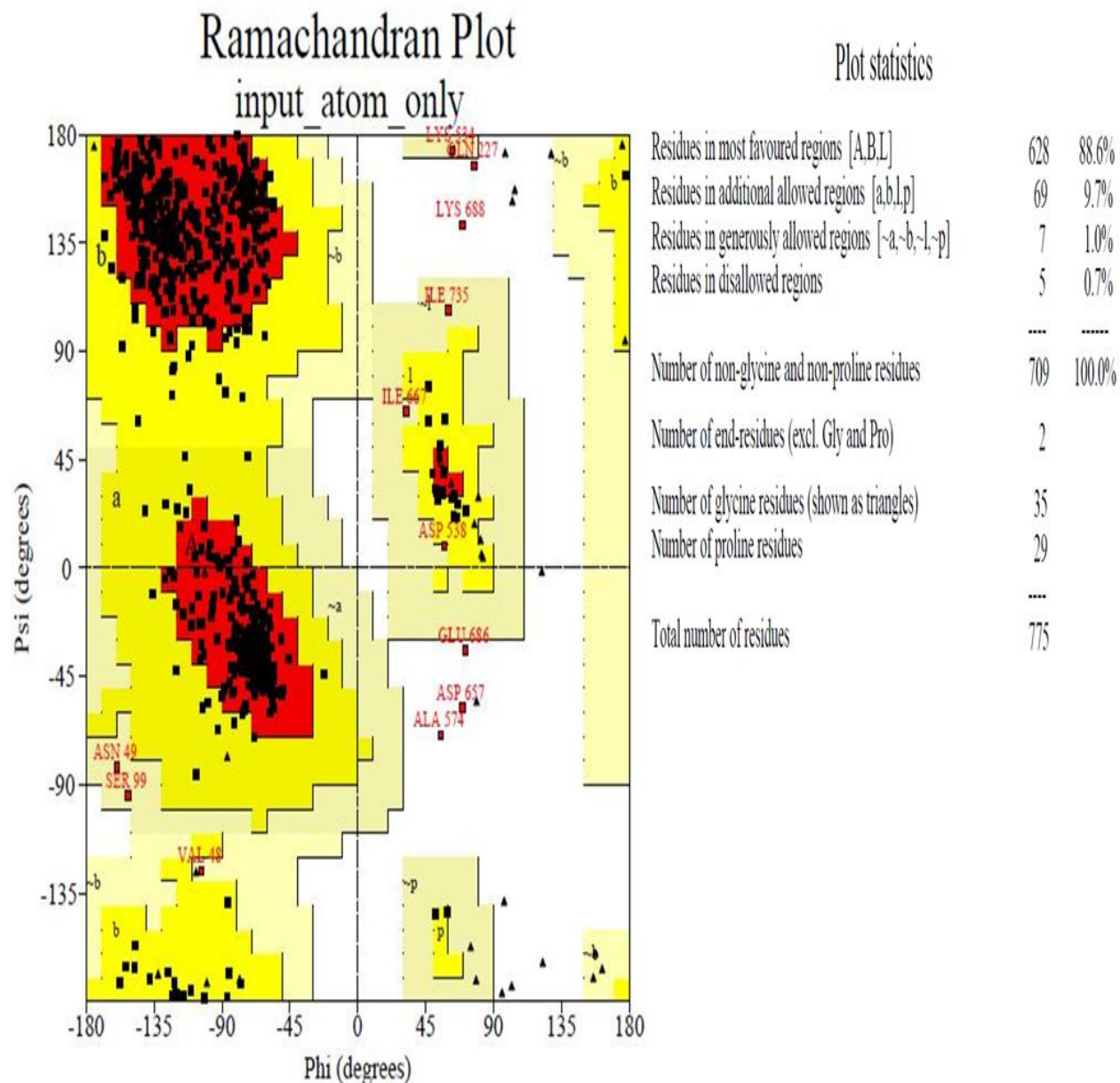

**S3 Fig: Ramachandron plot and its statistics of predicted VP4 3D model.**

Supplement: Supplementary file 4 [file Image3.PDF]

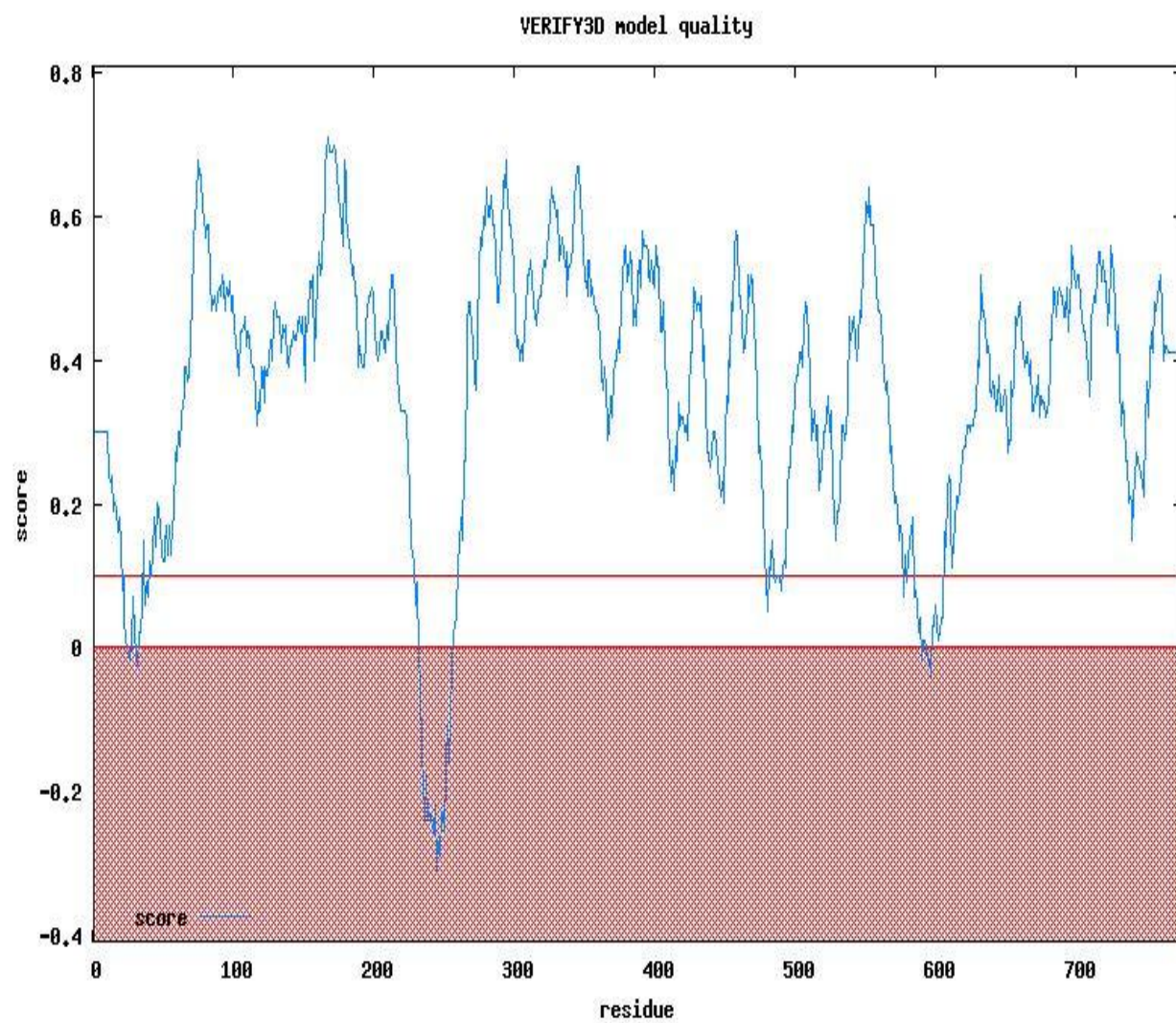

**S4 Fig: Quality assessment for predicted VP4 protein model.**

Supplement: Supplementary file 5 [file Image4.PDF]
