# Supplementary material for: Therapeutics Insight with Inclusive Immunopharmacology Explication of Human Rotavirus A for the Treatment of Diarrhea
Source: Front Pharmacol. 2016 Jun 23;7:153. doi: 10.3389/fphar.2016.00153 (PMC4917548; doi:10.3389/fphar.2016.00153)
Supplement: Supplementary file 6 [file Image5.PDF]

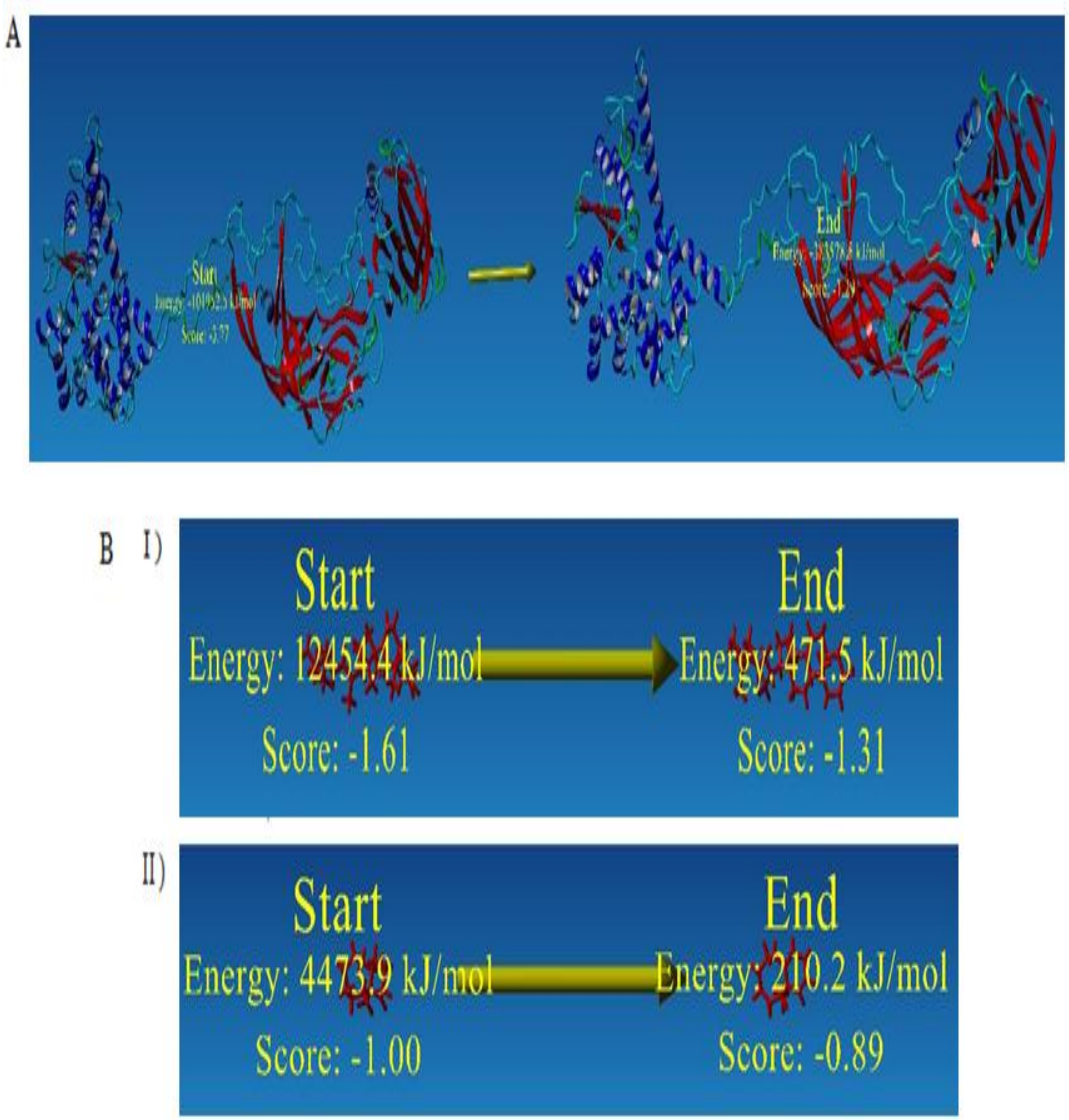


**S5 Fig: Energy minimized of 3D predicted model of VP4 Bangladeshi strain [A] and drawn drug molecule [B] I) Rhizophorine II) 1-Hydroxy-5 oxobicyclo[6.4.0]dodecane**
